# Modeling human migration across spatial scales in Colombia

**DOI:** 10.1371/journal.pone.0232702

**Published:** 2020-05-07

**Authors:** Amir S. Siraj, Alessandro Sorichetta, Guido España, Andrew J. Tatem, T. Alex Perkins

**Affiliations:** 1 Department of Biological Sciences, Eck Institute for Global Health, University of Notre Dame, Notre Dame, Indiana, United States of America; 2 School of Geography and Environmental Science, WorldPop, University of Southampton, Southampton, England, United Kingdom; 3 Flowminder Foundation, Stockholm, Sweden; University of Wisconsin Madison, UNITED STATES

## Abstract

Human mobility, both short and long term, are important considerations in the study of numerous systems. Economic and technological advances have led to a more interconnected global community, further increasing the need for considerations of human mobility. While data on human mobility are better recorded in many developed countries, availability of such data remains limited in many low- and middle-income countries around the world, particularly at the fine temporal and spatial scales required by many applications. In this study, we used 5-year census-based internal migration microdata for 32 departments in Colombia (i.e., Admin-1 level) to develop a novel spatial interaction modeling approach for estimating migration, at a finer spatial scale, among the 1,122 municipalities in the country (i.e., Admin-2 level). Our modeling approach addresses a significant lack of migration data at administrative unit levels finer than those at which migration data are typically recorded. Due to the widespread availability of census-based migration microdata at the Admin-1 level, our modeling approach opens up for the possibilities of modeling migration patterns at Admin-2 and Admin-3 levels across many other countries where such data are currently lacking.

## Introduction

Human mobility, both short- and long-term, are increasingly recognized as important drivers for many processes of societal importance including demographics, economics, regional development, and epidemiology. Human migration, along with births and deaths, determine population dynamics at both national and sub-national scales [1]. Macro- and micro-level economic studies have identified migration as a driver of labor flow [2], while human movement, along with movement of goods and ideas, has driven economic integration and development across regions [3,4]. Urban and regional development plans seek to meet growing demands for infrastructure and services with the aim of accommodating the movement of people and goods to new expansion regions [5]. Furthermore, as people move from place to place, they carry a multitude of infectious agents with them, enhancing the potential for increased disease transmission and enabling diseases to spread into new regions [6,7].

Human mobility has been modeled using a variety of data sources including census [8,9], road and air transport network [10,11], and mobile phone data [12,13]. Currently, large differences exist among countries regarding the availability of input data for modeling and better understanding human mobility across multiple temporal and spatial scales. Indeed, while there is an abundance of such data in high-income countries, they are either not available or difficult to access in many low- and middle-income settings. Census-based migration data, representing reliable proxies of the relative strength of short-term human mobility across multiple temporal scales [7,14], are often only available at rather coarse spatial scales. As a result, they are inappropriate for many applications, including modeling infectious disease dynamics, which require understanding of human mobility at much finer spatial scales. For example, the spread of Zika in Colombia shows much greater heterogeneity at the municipal level than at the departmental level [15,16] and thus understanding human mobility and the relative strength of connectivity at the department level in this setting is less than ideal for modeling Zika and other disease pathogen movements [17]. In this context, to enable the use of migration data available at a coarser spatial scale than the one matching the needs of the system of interest, inferential tools must be used to predict migration at finer spatial scales that can be used for better (i) understanding infectious disease dynamics, in relation to population movement, and (ii) supporting their control and elimination planning.

While migration may take place for a variety of reasons, it can be broadly generalized that people migrate seeking to maximize profits while minimizing costs [18]. Multiple studies have broken down the cost-benefit considerations of the push and pull factors into socio-demographic [19], socio-political [20], economic [21], geographic [22,23], and climatic and environmental factors [19,24]. Human migration has been modeled as a function of these factors with varying complexities mostly using intervening opportunity models [25,26], agent-based models [27,28], radiation models [29,30], and gravity models [31,32]. In particular, gravity-based spatial interaction models have been widely used in studying the flow of trades [33,34], labor [35,36], road traffic [37], communications [38], infections [39–41], and indeed human migration [8,42]. As it applies to human migration, the gravity model’s basic structure assumes that migration is proportional to the population size at the origin and destination, and inversely proportional to the distance between these two locations [43]. The basic form of gravity model with all exponents set to 1 has been widely used and subsequently extended to account for additional push and pull factors, including geographic, socio-demographic, economic, and environmental characteristics of the origins and destinations [19]. These advanced spatial interaction models have contributed greatly to better understand the absolute and relative importance of locations’ characteristics as measures of attractiveness and repulsiveness beyond their population size and distance [8,44]. These factors were chosen because it was demonstrated that they alone are able to explain most of the variance in gravity models of internal migration flows [8]. In particular, contiguity expected to have a positive impact on migration [8,19], proportion of urban population, tiny and major administrative units, and regional equivalent of the gross domestic product, which can all be considered proxy for economic opportunities, having different impacts on migration depending on their value at origin and destination [8,44].

In this study, we developed a novel spatial interaction modeling approach for Colombia using previously identified economic, socio-demographic and geographic factors including the regional equivalent of the gross domestic product, relative and absolute population size values, proportion of urban population, geographic contiguity of locations, and distance between locations. We used data collected at the admin-1 level (department) to fit a model that (a) estimates migration at the admin-2 level (municipality) and (b) aggregates those estimates back to Admin-1 level to calculate likelihood for selecting the best performing model. We also used migration data collected at intermediate level that includes data for geographic units ranging from single municipalities to multi-departments, with a subset of the data (single municipality) used to validate our model (please refer to Data and Method section for details). We used a logistic regression model with coefficients estimated using Markov Chain Monte Carlo (MCMC), a Bayesian approach to statistical inference. Our modeling approach (i) addresses the current lack of migration data either not collected or not available at a sufficiently fine spatial scale, with potential application in many countries, and (ii) further provides a novel framework for using coarse migration data to predict migration at finer spatial scales.

## Data and methods

### Migration data

We extracted census-based internal migration data for Colombia from the most recent census microdata available through the online Integrated Public Use Microdata Series-International (IPUMSI) database [45]. These data are based on a 10-percent sample of the whole 2005 census and contain information about the department of residence of the respondents both in the census year and five years prior to the census. The data served as a proxy for the number of migrants to and from all Colombian departments (n = 33), while data pertaining to two among them (i.e., Vaupes and Guainía) were combined in the census year, yielding in 32 source and destination geographic units.

The IPUMSI data also included migration data for 533 census units, which represent single municipalities and groups of contiguous municipalities with population above 20,000 in 1993. (https://international.ipums.org/international-action/sample_details/country/co#co2005a). Since the groups of municipalities that were used to identify the place of origin do not spatially match those used to identify the place of destination, we merged together some of the contiguous groups of municipalities and aggregate the associated migration information, to make the two sets of geographic regions similar. This resulted in 276 uniquely identifiable and temporally consistent census units, of which 147 were single municipality and 129 were multi-municipality units. This also meant 5% of all migration routes between the 276 locations (i.e. origin and destination pairs) were within the same department, representing intra-departmental migration flows.

### Population data

We used the Gridded Population of the World (GPWv4) population count dataset [46] referring to the year 2005 and having a spatial resolution of 30 arc-seconds (approximately 1km at the equator). We extracted the population figures for the 32 departments and 1,122 municipalities in Colombia using the corresponding departmental and municipal administrative boundary shapefiles obtained from the National Geographical Information System of Colombia [47]. We also used the 2000/2001 MODIS 500 m Global Urban Extent dataset [48], having a resolution of 15 arc seconds (approximately 500 m at the equator), to estimate the proportion of urban population in each department, multi-municipality unit, and municipality.

### Economic data

To account for socioeconomic differences between administrative units with potential effect on human migration flows, we used the G-Econ (4.0) dataset (Nordhaus, 2006), that provides gridded Purchasing Power Parity (PPP) adjusted Gross Cell Product (GCP) for 2005, with a resolution of 1-degree (~ 111 km at the equator). To express the gridded PPP values on a per capita basis, we divided them by the corresponding gridded population; with the latter derived from the 2005 Gridded population count dataset [49]. We chose this gridded population data as it was originally used to calculate the 2005 gridded PPP values (Nordhaus, 2006). Grid cells with missing GCP values were imputed with the mean of the surrounding eight grid cell values. Once we obtained a complete layer at one-degree resolution, we resampled the layer, without smoothing, to a resolution of 2.5 arc-minutes (~5km), and extracted average values at department, multi-municipality unit, and municipality levels.

### Geographic data

QGIS software [50] was used to calculate Euclidean distances among population-weighted centroids of each department, multi-municipality unit and municipality. This was done after projecting the corresponding shapefiles to a customized projected coordinate system to minimize linear distortion within the study area. To record the contiguity between each spatial unit in the three groups listed above, we generated a binary variable with a value of 1 if two units share a boundary and of 0 otherwise.

### Fitting a novel spatial interaction model

Given that the migration data extracted from the IPUMSI database are based only on a 10 percent sample of the whole census, we used a logistic regression to model the proportion of people migrating between departments during the 5-year timespan (i.e., 2000–2005). For ease of interpretation, we transformed the data to account for proportion of people who moved from department *I* (source) to department *J* (destination), while the IPUMSI data referred to the number of people in department *J* who moved from department *I* over the five year time frame. The model we used to estimate migration at finer spatial scale (Admin-2) based on observations at coarser scale (Admin-1) follows a binomial model as described as follows:

$$Y_{IJ}\sim binomial\left(N_{I},P_{IJ}\right)$$ (1)

$$P_{IJ}=\frac{\Sigma_{i}\Sigma_{j}N_{i}v_{ij}}{\Sigma_{i}\Sigma_{j}N_{i}}$$ (2)

$$logit\left(v_{ij}\right)=\beta_{0}+\beta_{1}d_{ij}+\beta_{2}N_{i}+\beta_{3}N_{j}+\Sigma_{k=4}^{n}\beta_{k}X_{k}$$ (3)

where *Y*_*IJ*_ is the observed number of people who migrated from department *I* to *J*, *N*_*I*_ is the population in department *I*, *P*_*IJ*_ is the estimated proportion of people in department *I* who migrated to department *J* based on aggregated municipality level migration estimates.

**Fig 1:**
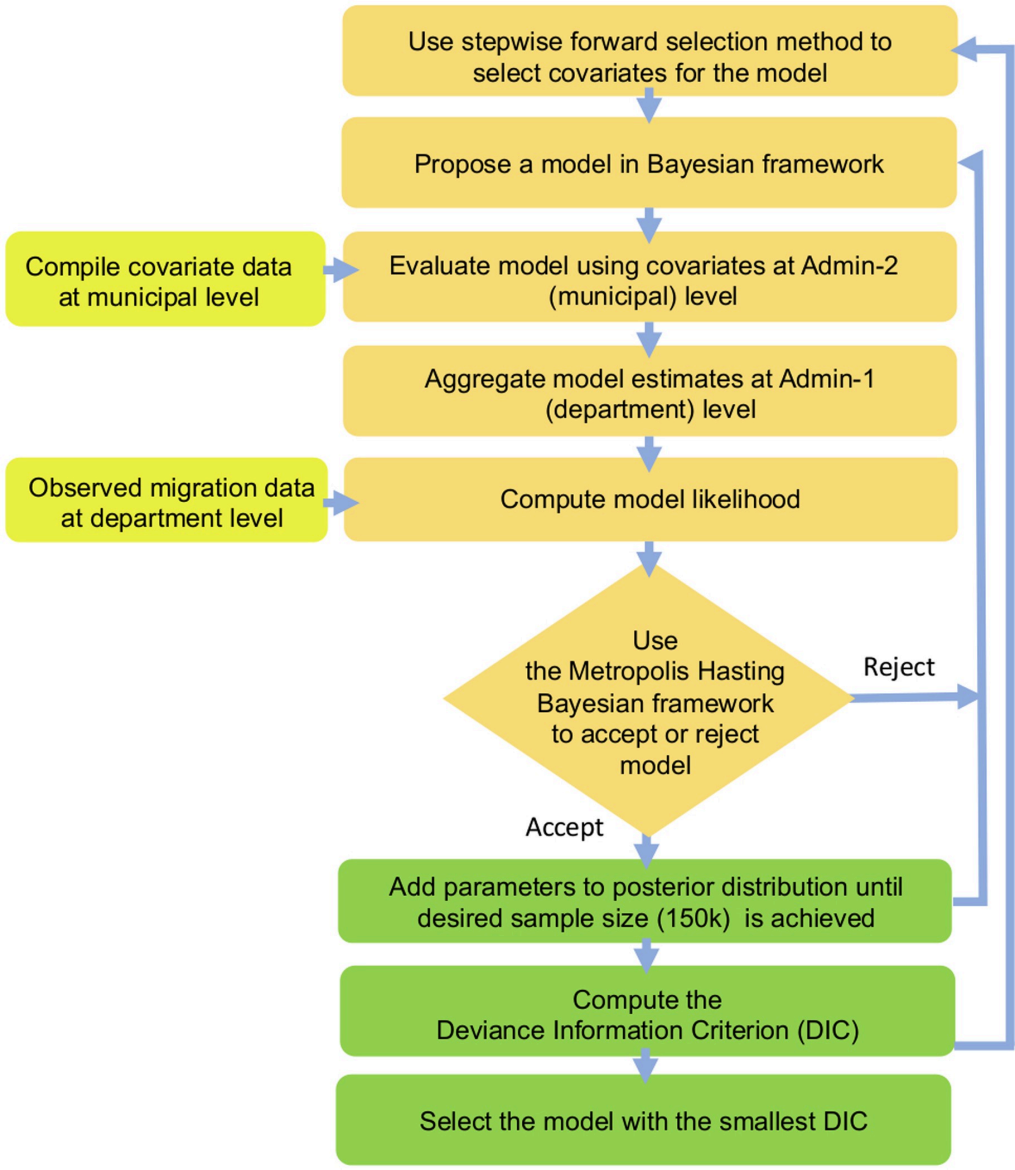
Flowchart of the Bayesian model selection process under the fine-scale model approach. The input stages are shown in yellow, and the processing stages are shown in orange, while the output stages are in green.

Our model selection approach, which we termed as fine-scale approach, involved an aggregation step described in Eq (2), where we convert model estimated migration at a finer spatial scale (Admin-2) into proportions at a coarser spatial scale (Admin-1) (Fig 1). Accordingly, we first predicted *v*_*ij*_, the proportion of migrants from each municipality *i* in the department *I* to each municipality *j* in department *J*, using Eq (3), where *β*_*0*_,…,*β*_*n*_ are regression coefficients, *d*_*ij*_ is the distance between the municipalities of origin *i* and destination *j*, *N*_*i*_ and *N*_*j*_ represent the total population at the origin and destination municipalities respectively, and *X*_*k*_ are the covariates used in the model. Second, we estimated *P*_*IJ*_, the proportion of people in department *I* who migrated to department *J*, using Eq (2), where *N*_*i*_ is the population in each municipality *i* in the department *I*.

### Likelihood function

Our selection of the best model relies on the quantity that we calculate to compare goodness of fit for each model, in our case the likelihood, i.e. the probability of the model given the observed data–expressed in log scale for computational convenience. Because our final goal was to predict municipality level migration proportions, we aggregated the migration proportions to their respective departments of origin *I* and destination *J*, which we refer to as *P*_*IJ*_. To enable fitting the aggregated proportion *P*_*IJ*_ at the department level, we defined the log-likelihood of the model coefficients as:

$$LL=\sum_{I}\sum_{J}M_{IJ}\;ln\;ln\left(P_{IJ}\right)+\left(N_{I}-M_{IJ}\right)ln\left(1-P_{IJ}\right)+K$$ (4)

for *I ≠ J*, where *M*_*IJ*_ is the census-based number of people in department *I* who moved to department *J*, *N*_*I*_ is the population size at the source department *I*, and a constant $K=lnln\left(N_{I}!\right)-lnln\left(M_{IJ}!\right)-ln\left(\left(N_{J}-M_{IJ}\right)!\right)$.

### Coefficient estimation

We used the Bayesian Tools package in R [51] to estimate coefficients based on the Markov Chain Monte Carlo (MCMC) Bayesian approach implemented according to the Metropolis-Hastings algorithm [52,53]. Parameters estimated in the models (i.e., coefficients of the logistic regression models) include distance between origin and destination, population at the origin and destination, and additional covariates included using a stepwise forward model selection approach (Table 1). Prior to fitting the model, we scaled all continuous explanatory variables to obtain a mean of 0 and standard deviation of 0.5. Categorical binary variables were transformed to have a mean of 0 and a range of 1 [54]. This process helped stabilize parameter estimates for some of the large-value variables (e.g., population and distances) and resulted in model coefficients that could be compared across variables. For each coefficient, we assumed a Cauchy prior distribution centered at 0 with a scale parameter value of 2.5, except for the intercept, which was set to have a scale parameter value of 10. This assumes that extremely large coefficients (greater than 5 in logistic regression for centered variables) are highly unlikely [54].

**Table 1:** List of normalized covariates used in selecting our migration model and their corresponding distributions.

Variable name	Description
***DIST***_***ij***_	Distance between the population-weighted centroids of the origin and the destination municipalities (in km)
***POP***_***i***_	Population of origin municipality
***POP***_***j***_	Population of destination municipality
***GECON***_***i***_	Average per-capita gross cell product of origin municipality
***GECON***_***j***_	Average per-capita gross cell product of destination municipality
***TINY***_***i***_	= 1 if population of origin municipality < 10th percentile; 0 otherwise
***TINY***_***j***_	= 1 if population of destination municipality < 10th percentile; 0 otherwise
***URBANPROP***_***i***_	Proportion of urban population in the origin municipality
***URBANPROP***_***j***_	Proportion of urban population in the destination municipality
***PERC***_***i***_	Percentile of the origin population among all municipalities
***PERC***_***j***_	Percentile of the destination population among all municipalities
***MAJCEN***_***i***_	= 1 if population of origin municipality > 90th percentile; 0 otherwise
***MAJCEN***_***j***_	= 1 if population of destination municipality > 90th percentile; 0 otherwise
***CONT***_***ij***_	= 1 if the origin and destination municipalities share common borders; = 0 otherwise

### Model selection

We used a stepwise forward selection to identify the best predictive model with the lowest Deviance Information Criterion (DIC), starting with the basic gravity model, which accounts only for distance between origin and destination, and their population. For each candidate model, we ran the MCMC procedure five times, each from different initial conditions, which enabled us to assess convergence of the parameter values and calculate the DIC more robustly. Accordingly, each chain was run for 1.5 x 10^5^ steps, with the first half constituting the burn-in that we excluded from our posterior distribution. Our posterior distribution was then generated from the remaining MCMC samples by thinning every 10 steps in each chain. All parameter initializations and proposals were done using the Bayesian Tools package in R [51], while convergence of parameter assessed using the Gelman-Rubin diagnostic [55].

**Fig 2:**
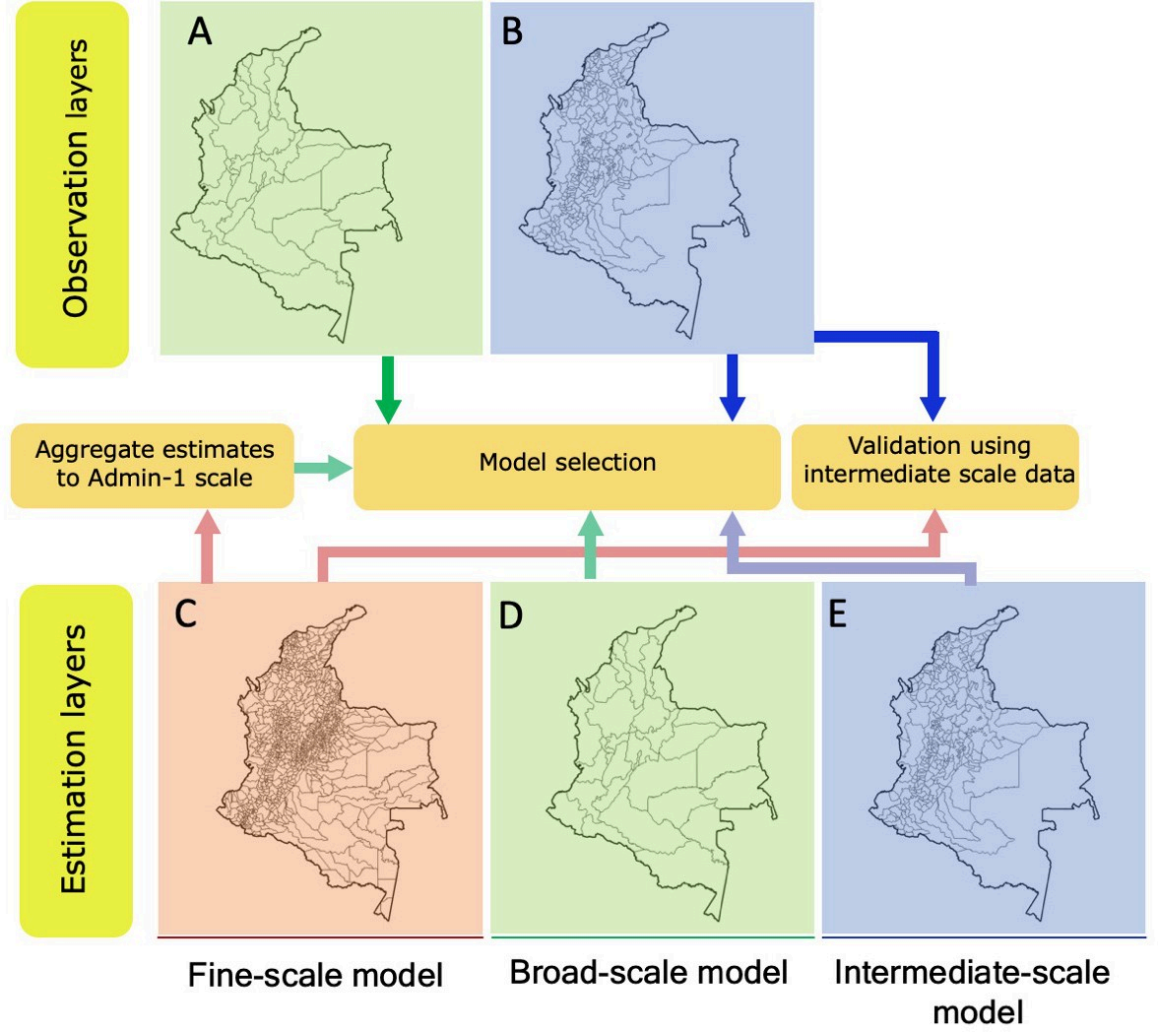
Schematic of the three spatial interaction modeling approaches and subsequent validation. In the first approach (fine-scale), model coefficients are estimated at the municipality level (n = 1122) (C) and used to estimate municipality level migrations, which are subsequently aggregated to the corresponding departments of origin and destination. The estimated department level flows are then used to calculate likelihood (select the best model) based on the observed migrations at the department level (n = 32) (A). In the second approach (broad-scale), model coefficients are estimated at the department level (n = 32) (D) yielding in predicted migration proportions which are used to calculate the likelihood based on observed department level migrations (A). In the third approach (intermediate-scale), model coefficients are estimated at the intermediate level (n = 276) (E), migration proportions are predicted at the intermediate level, and likelihood calculated based on observed intermediate level migrations (n = 276, including 147 single municipality units) (B). To validate our fine-scale model estimates, we used a subset of the intermediate level observation (B) that only includes migrations to and from single-municipality units. Bold lines represent observed data, while deemed lines represent estimates corresponding to each approach namely: fine-scale (red), broad-scale (green) and intermediate (blue).

We tested three different spatial interaction modeling approaches: (1) the fine-scale model at the municipality level fitted to observed data available at department level, with migration proportions predicted at the municipality level (Fig 1), (2) a broad-scale model at the department level fitted to observed data available at the department level, with proportions predicted at the same level, and (3) an intermediate-scale model (i.e., based on the 147 municipalities and 129 multi-municipality units) fitted to observed data available at the same intermediate level, with proportions predicted at the intermediate level (Fig 2).

### Model validation

To validate our fine-scale model results obtained in the absence of observed municipality level migration data, we used the observed data available at the intermediate level, based on 276 census units including 147 single municipalities and 129 multi-municipalities extracted from the 2005 census (Fig 1B). We used the best model selected at the municipality level (i.e., fine-scale model) to predict migration proportions at the municipality level, converted them to municipality level migration flows and aggregate the latter to the corresponding intermediate level, and finally compared the resulting estimated intermediate level migration flows to the corresponding observed flows derived from the 2005 census. To compare the intermediate level migration flows with those we would expect based on the broad-scale model, we assumed that migration proportions predicted at the department level can be uniformly disaggregated within each department and thus assigned the same proportion to all single municipalities and multi-municipalities located within each department.

## Results

### Fine-scale model

Our best fine-scale model, selected using the forward-selection had all covariates related to the destination significantly associated with migration, except for percentile of population (*PERCj*) and the economic status (*GECONj)* (Table 2). Urban proportion (*URBANPROPi*) and economic status (*GECONi)* were the only origin-related covariates with a significant contribution towards migration (p-value <0.001), along with the origin’s population size (Table 1). All coefficients in the best model showed convergence in our diagnosis based on the Gelman-Rubin diagnostic [55], all having potential scale reduction factor of approximately 1.0 (S1 Fig).

**Table 2:** Iterative model selection based on Deviance Information Criterion (DIC) using forward step-wise inclusion for fine-scale models, with a single variable added at each iteration.

Model	Explanatory variables	DIC
**Iteration 1**	*DIST*_*ij*_, *POP*_*j*_, *POP*_*i*_	112926.5
**Iteration 2**	*DIST*_*ij*_, *POP*_*j*_, *POP*_*i*_, *URBANPROP*_*j*_	109357.0
**Iteration 3**	*DIST*_*ij*_, *POP*_*j*_, *POP*_*i*_, *URBANPROP*_*j*_, *URBANPROP*_*i*_	104652.7
**Iteration 4**	*DIST*_*ij*_, *POP*_*j*_, *POP*_*i*_, *URBANPROP*_*j*_, *URBANPROP*_*i*_, *MAJCEN*_*j*_	10107.87
**Iteration 5**	*DIST*_*ij*_, *POP*_*j*_, *POP*_*i*_, *URBANPROP*_*j*_, *URBANPROP*_*i*_, *MAJCEN*_*j*_, *CONT*_*ij*_	98642.46
**Iteration 6**	*DIST*_*ij*_, *POP*_*j*_, *POP*_*i*_, *URBANPROP*_*j*_, *URBANPROP*_*i*_, *MAJCEN*_*j*_, *CONT*_*ij*_, *GECON*_*i*_	96959.43
**Iteration 7**	*DIST*_*ij*_, *POP*_*j*_, *POP*_*i*_, *URBANPROP*_*j*_, *URBANPROP*_*i*_, *MAJCEN*_*j*_. *CONT*_*ij*_, *GECON*_*i*_, *TINY*_*j*_	56363.93

**Fig 3:**
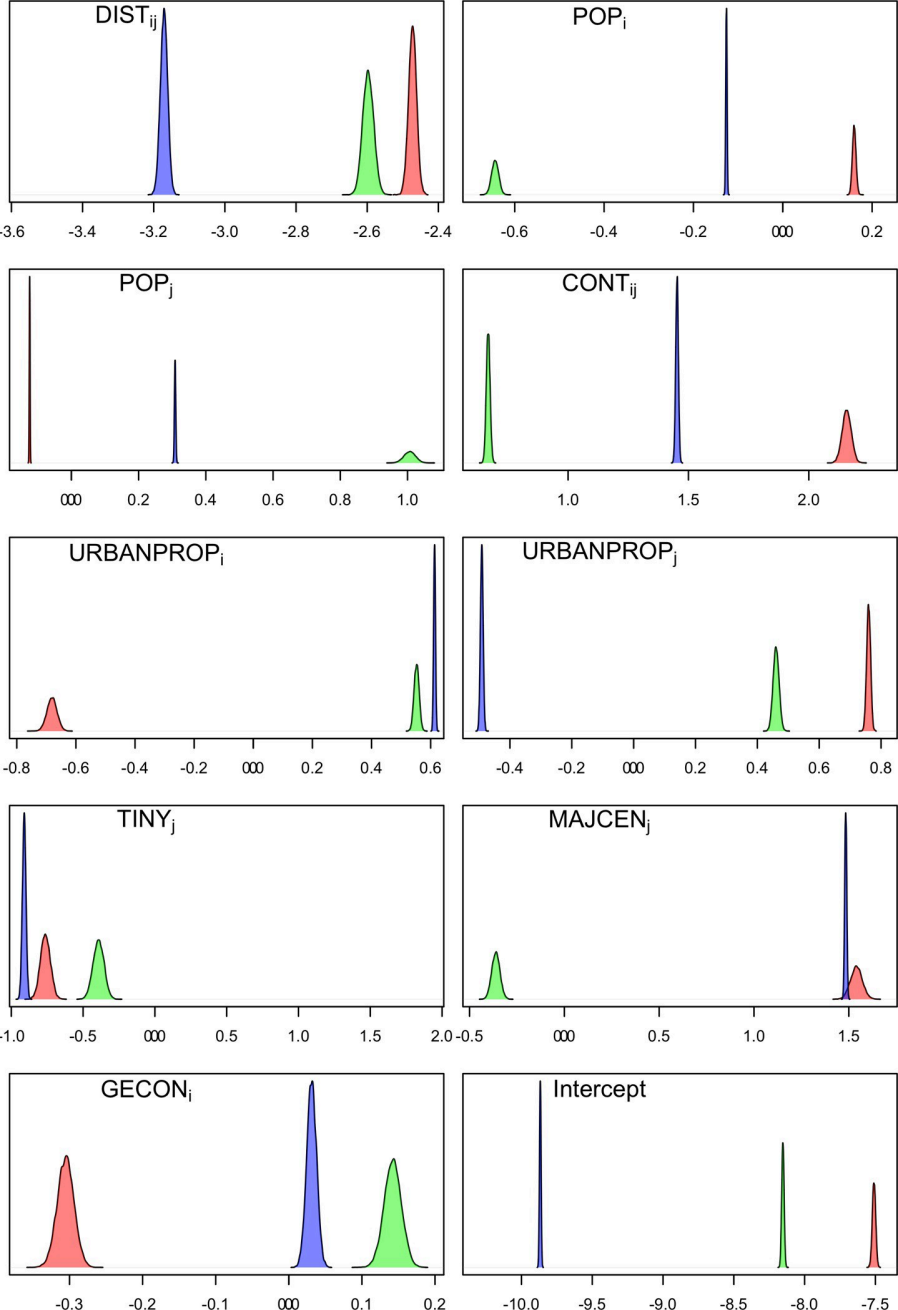
Posterior distribution of coefficients selected based on the best fine-scale model (red) compared to parameter values we would get when fitting using the broad-scale modeling approach while covariates are restricted to those selected in the best fine-scale model (red); and compared to parameter values we would get when fitting using the intermediate-scale modeling approach while covariates are restricted to those selected in the best fine-scale (blue). All coefficients were significant at least at the 0.05 level.

The best fine-scale model showed that distance between origin and destination was the most important covariate with the highest negative effect, with a coefficient value of -2.47 (95% CI: -2.49 − -2.45) (Table 3). Contiguity (*CONTij*) and the destination’s status of being a major population center (*MAJCENj*) had the second and third largest positive contributions towards migration, having coefficients of 2.16 (95% CI: 2.12−2.19) and 1.54 (95% CI: 1.48 − 1.6), respectively. Urban proportion at destination and origin were also important, with coefficient of 0.76 (95% CI: 0.75 − 0.77) and -0.68 (95% CI: -0.71 − -0.65) respectively, suggesting mostly-urban municipalities as preferential destinations and mostly-rural municipalities as sources of migrants. Not only being a mostly rural municipality but representing a tiny population center as well was associated with generating migrants, with a coefficient of -0.76 (95% CI: -0.83− -0.7). Population sizes had mixed effects on migration, with the origin population (*POPi*) showing a positive effect with a coefficient of 0.16 (95% CI: 0.15 − 0.17) and the destination population (*POPj*) showing a negative effect with a coefficient value of -0.124 (95% CI: -0.126 − -0.122) (Fig 3, in red). Our results of significant effect of both distance and contiguity between origin and destination and the opposing effects of urban proportion at origin and destination, as well as the effect of poor economic activities at the origin, suggest that urban economic centers are more likely to attract migrants from close, rural municipalities with low economic activity. Selected covariates and coefficient values for the best models under the broad-scale and the intermediate-scale approaches are shown in Supporting Information (S3 and S4 Tables).

**Table 3:** Coefficients of the best fine-scale model.

Covariate		Median	95% CI*	R-hat
**Distance between origin and destination**	*DIST*_*ij*_	-2.472	[-2.495, -2.451]	1.000
**Population of origin**	*POP*_*i*_	0.16	[0.152, 0.168]	1.000
**Population of destination**	*POP*_*j*_	-0.124	[-0.126, -0.122]	1.000
**Contiguity of origin and destination**	*CONT*_*i*_	2.157	[2.121, 2.194]	1.000
**Urban proportion of origin**	*URBANPROP*_*i*_	-0.681	[-0.715, -0.648]	1.000
**Urban proportion of destination**	*URBANPROP*_*j*_	0.76	[0.747, 0.772]	1.000
**Population of destination <10th percentile**	*TINY*_*j*_	-0.763	[-0.83, -0.696]	1.000
**Population of destination > 90th percentile**	*MAJCEN*_*j*_	1.54	[1.48, 1.601]	1.000
**Average per-capita gross cell product of origin**	*GECON*_*i*_	-0.305	[-0.328, -0.283]	1.000
**Intercept**		-7.51	[-7.53, -7.487]	1.000

* Credible Intervals (CI) obtained from the 2.5% and 97.5% quantiles of each parameter’s distribution.

### Model comparison across spatial scales

To further examine differences with respect to determinants of migration across spatial scales, we used the structure of the best-fit fine-scale model to (a) fit a model based on the broad-scale approach and (b) fit a model based on the intermediate-scale approach. Our results show that the models based on the broad- and intermediate-scale approaches, while having few similarities in terms of magnitude and sign of the coefficients, have several differences compared to the fine-scale model. For instance, there were differences between the fine-scale and broad-scale models in the magnitudes and signs of the coefficients for *POPi*, *POPj*, *URBANPROPi*, *MAJCENj* and *GECONi*, and in the magnitudes of the coefficients for *DISTij* and *CONTij* (Fig 3). In contrast, while there were large differences between the fine-scale and intermediate-scale models in the magnitude of coefficients for *DISTij*, *CONTij*, *URBANPROPi*, *URBANPROPj* and *POPi*, they only showed a sign difference in the coefficients for *TINYj and GECONi*. These results suggest that while migration at the three spatial levels exhibit different characteristics, as expected, the fine-scale migration patterns are relatively more similar to those at the intermediate scale and less similar to those at the broad scale.

Overall, fine-scale model covariates that showed more similarity when applied to the intermediate-scale than to the broad-scale modeling approach included (a) factors that, according to our model, encouraged emigration such as contiguity between the origin and destination, origin’s higher population size, lower urban proportion and poorer economic status, and (b) factors that encouraged immigration such as the destination’s low population size, and being a major population center. Distance between origin and destination and urban proportion at destination were the only two fine-scale factors that showed more similarity when applied to the broad-scale than with the intermediate-scale modeling approach, suggesting that distance and urbanization are more robust to differences in scale than the other variables.

### Comparison of estimated municipality level migration flows based on the fine-scale and broad-scale model

**Fig 4:**
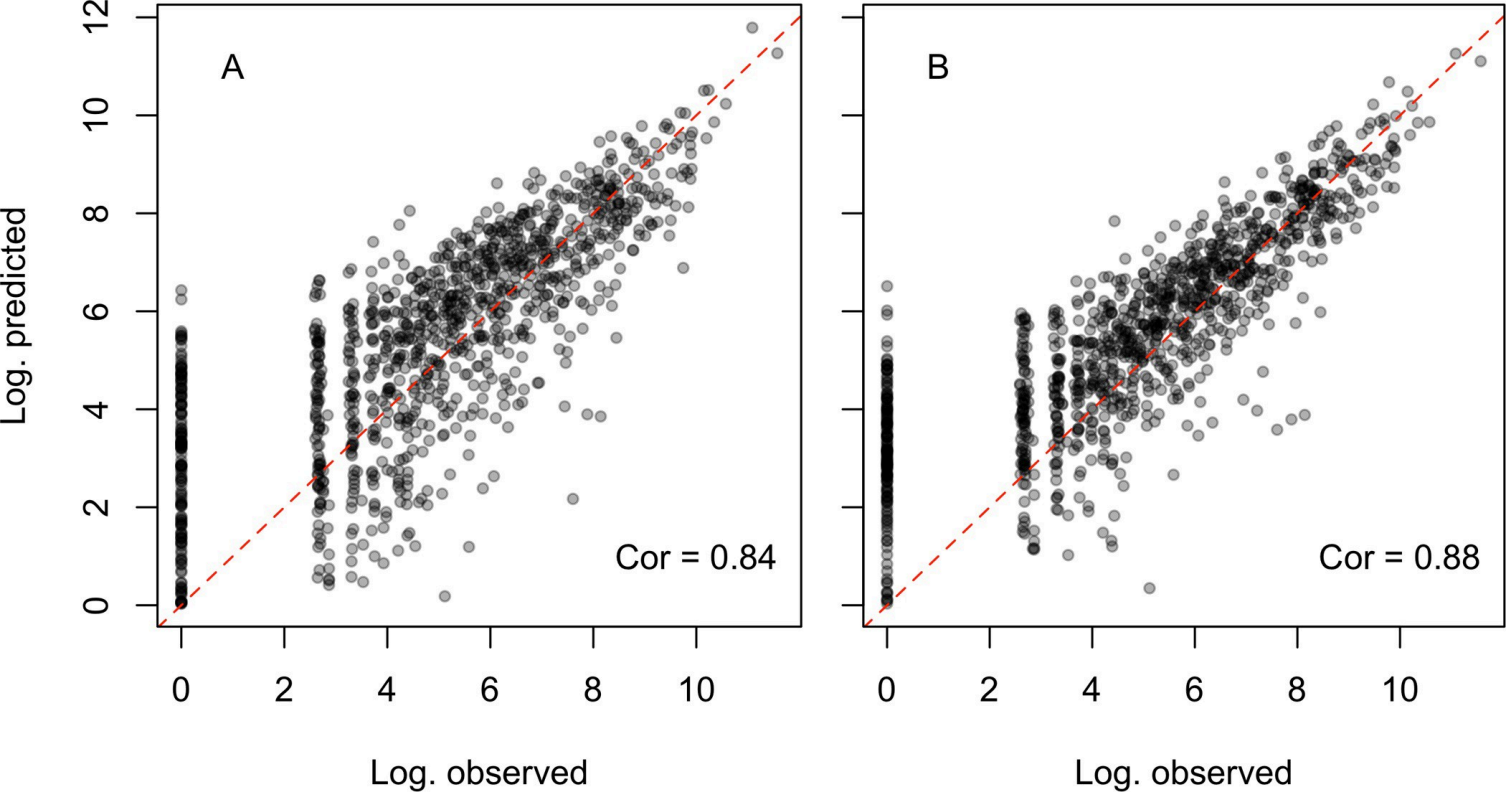
Estimated versus observed migration flows (in log scale) between each pair of departments with estimated flows (A) based on the results of the fine-scale model, aggregated to the department level, and (B) based on the results of the broad-scale model.

**Fig 5:**
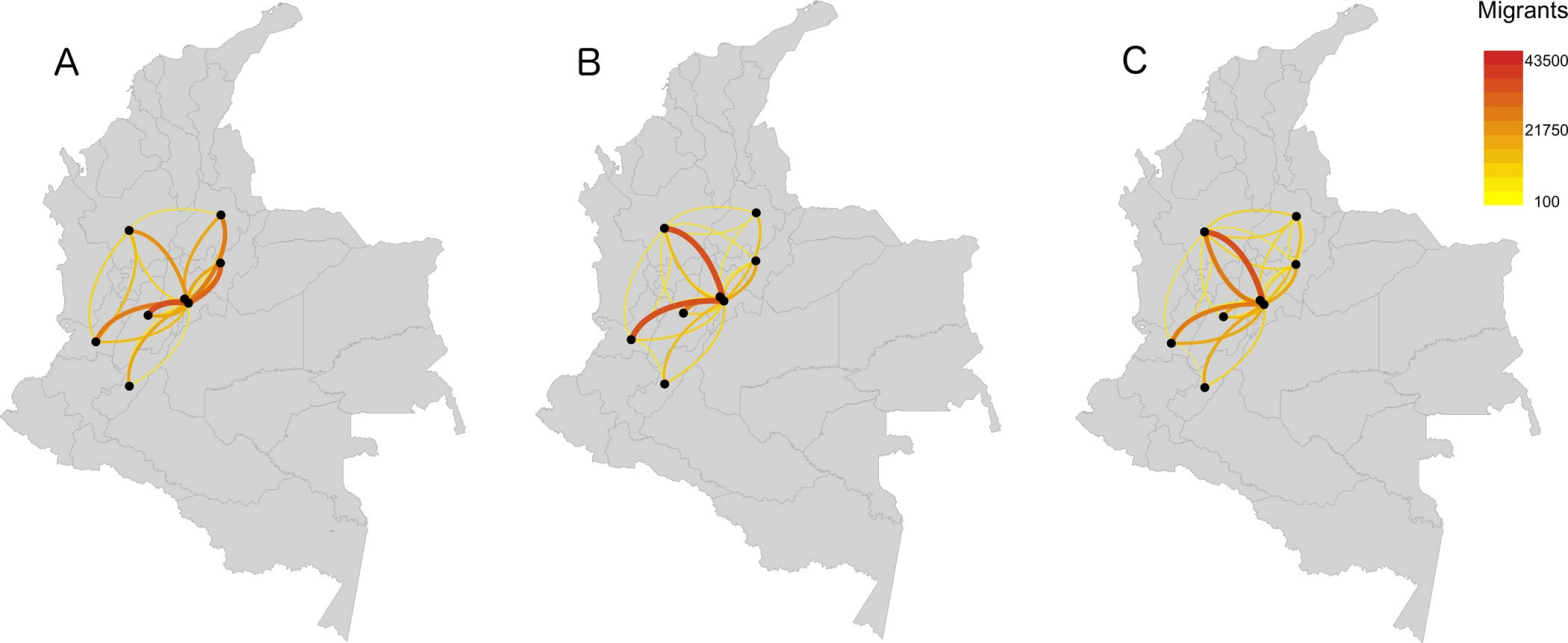
Observed (A) and estimated (B and C) migration flows between eight selected departments having the highest observed migration flows either as destination or origin. Estimated flows are (B) based on the results of the fine-scale model, aggregated to the department level, and (C) based on the results of the broad-scale model. Centroid points are weighted by the spatial distribution of population within each department.

Our estimated migration flows between any pair of municipalities, based on the fine-scale model, were aggregated to the department level and compared to the observed department level migration flows derived from the 2005 census. This resulted in a Pearson’s correlation coefficient of 0.84, as compared to 0.88 based on the results of the broad-scale model. These results demonstrate a good fit, comparable to those we would get based on the best broad-scale model (Fig 4), especially for routes with high observed migrations (Fig 5).

**Fig 6:**
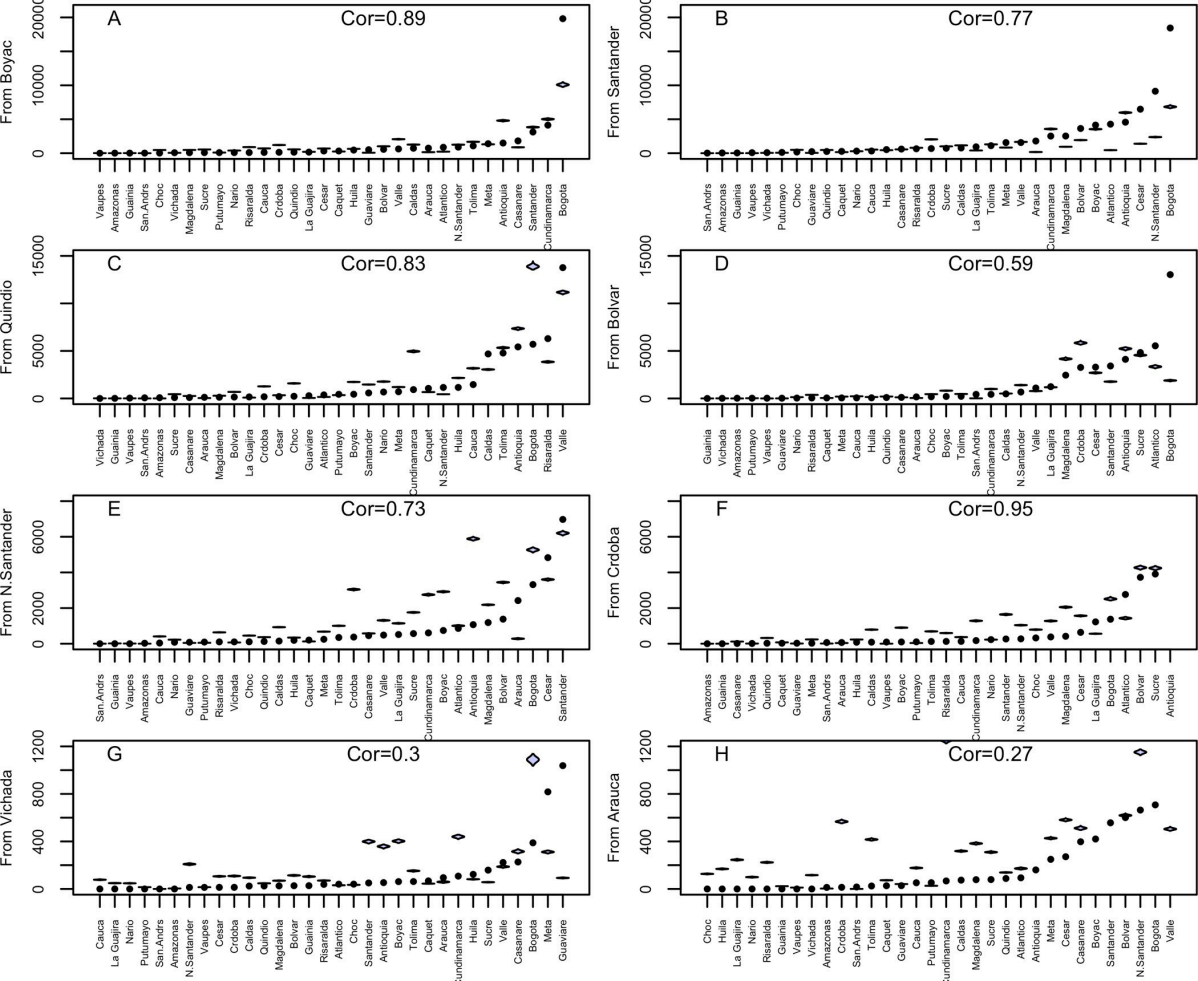
Predicted versus observed migration flows (sorted by magnitude) in eight departments characterized by (A & B) high, (C & D) medium, (E & F) low, and (G & H) very low incoming migration flows. The shaded violin plots show the 95% confidence interval based on the joint posterior sample parameters, while the black dots represent the observed flows. The four categories were selected based on the maximum number of migrants each department would receive based on our estimates, with each of the four departments selected randomly from each quartile.

We further compared the estimated migration flows aggregated to the department level to the corresponding observed flows to all possible destinations. Our results showed a good fit when compared to the observed migration flows, especially for departments characterized by relatively higher incoming flows (Fig 6A–6C). For departments characterized by relatively lower incoming flows, our results seem to overestimate migration (Fig 6D).

**Fig 7:**
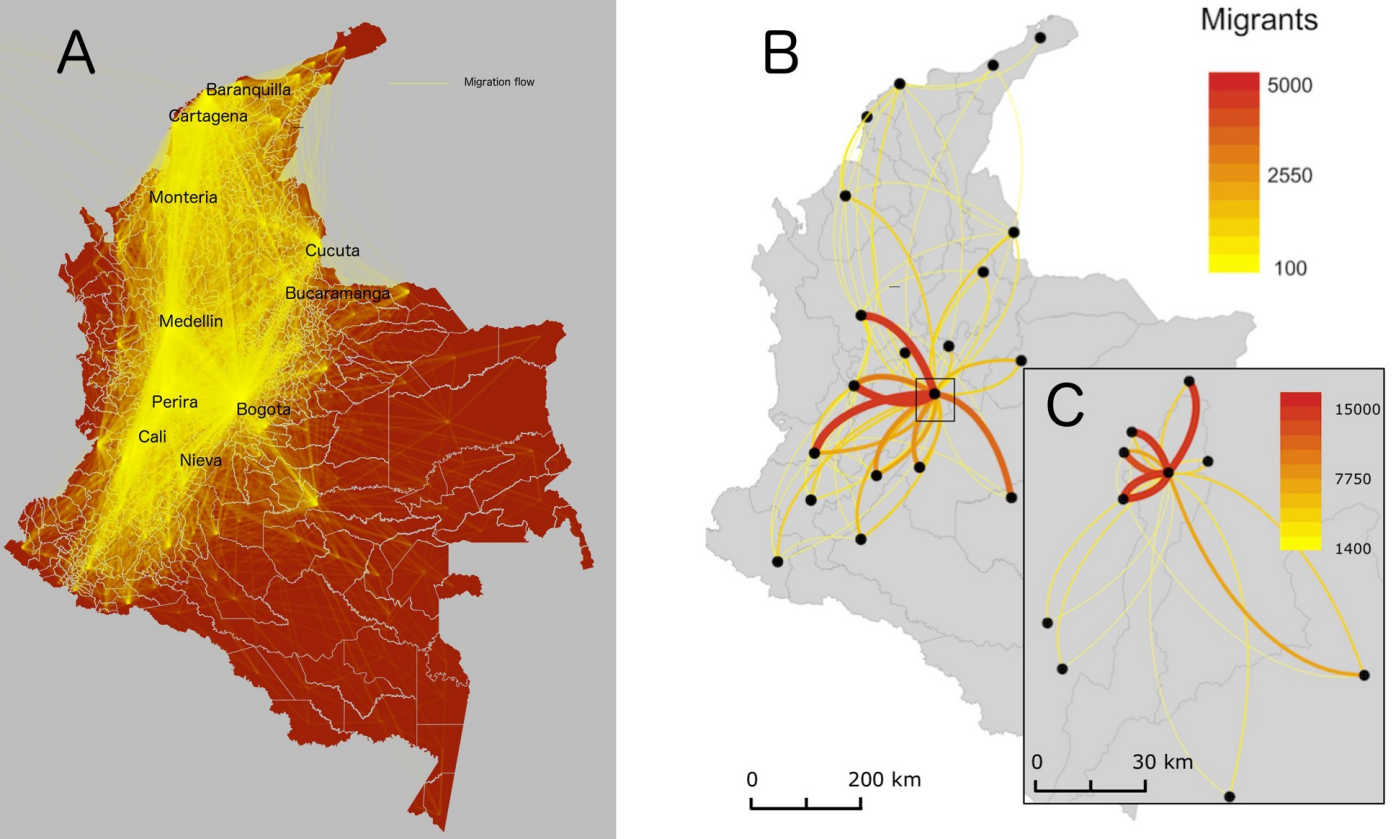
Estimated migration flows (A) between each pair of municipalities with lines going in both directions, (B) between municipalities that have the 20 highest migration flows and have distances of more than 100 km from each other, and (C) between municipalities that have the 10 highest migration flows and are within 100 km from Bogota. Centroid points are weighted by the spatial distribution of population in each municipality.

Our municipality level migration flow estimates, based on the fine-scale model, show a pattern of migration into major municipalities in Colombia including Bogota, Cali, and Medellin. However, there are also other significant migration routes to other relatively smaller municipalities in departments with relatively higher economic activity, such as Pereira in the department of Risaralda, or regional economic centers, such as Neiva in the department of Huila, Cucuta in the department of North Santander, and Monteria in the department of Cordoba (Fig 7A and 7B). These results further demonstrate the importance of urban centers and the economic opportunities they present as the main pull factors driving migrations at the municipality level. Note that similar patterns were observed at the department level, with those including major urban municipalities and representing economically strong departments attracting the largest share of migrants across Colombia (Fig 5A and 5B).

### Validation of the fine-scale model using intermediate level migration flows

**Fig 8:**
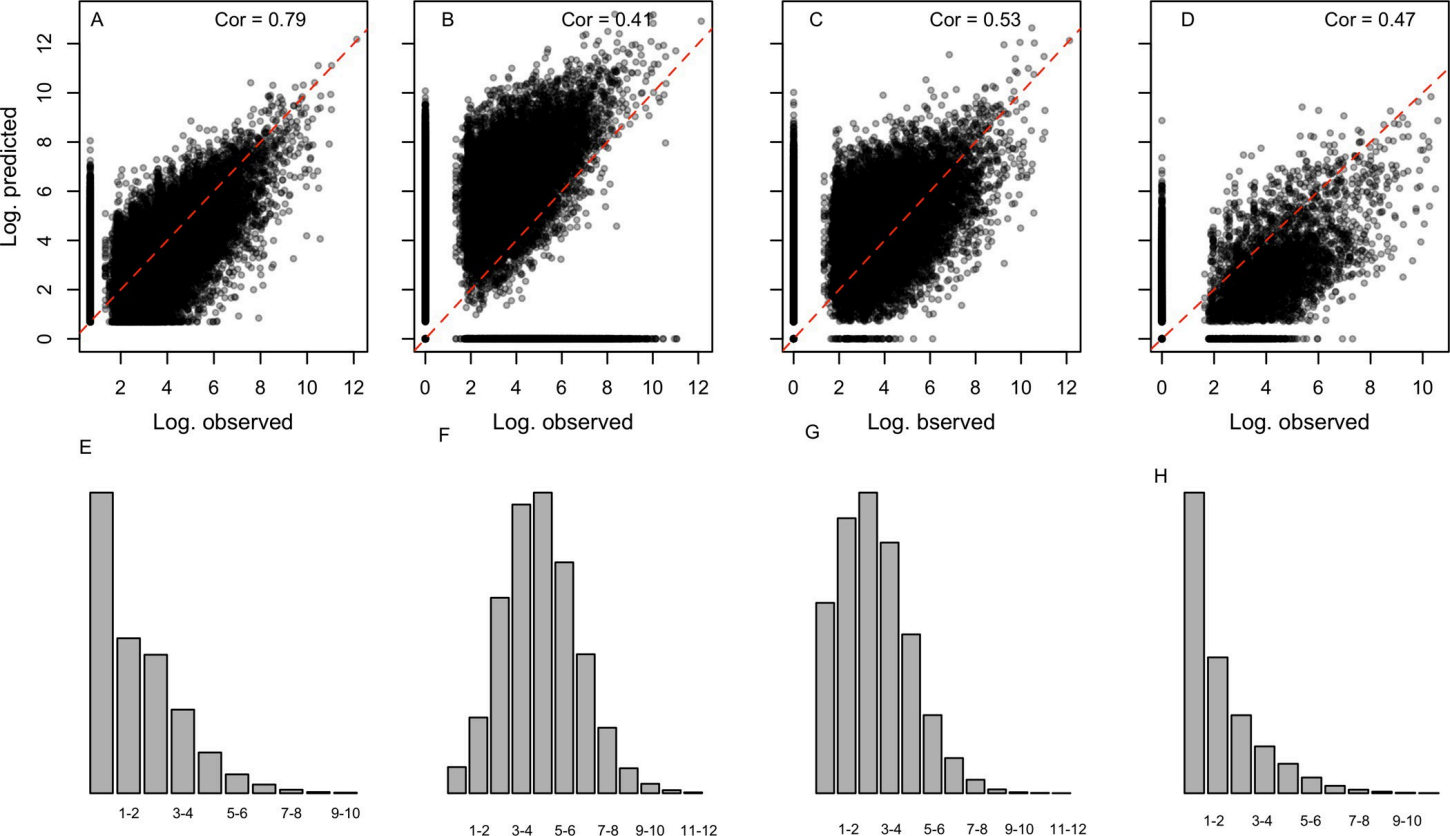
Estimated versus observed migration flows (in log scale) between each pair of intermediate level census units, with estimates based (A) on the intermediate-scale model using the intermediate level migration data, (B) on the broad-scale model by assigning the predicted department level migration proportions to the corresponding single municipalities and multi-municipalities, (C) on the fine-scale model by aggregating the estimated municipality level migration flows to the corresponding intermediate level census units, and (D) on the fine-scale model as in C, but considering only single-municipalities. Red lines represent the identity line, while E through H show histograms of the residuals (in log scale) for the corresponding plots in A-D.

Observed intermediate level migration flows, pertaining to 276 census units (please refer to Data and Methods section), were compared to estimated migration flows obtained by aggregating the fine-scale model estimates as well as disaggregating the broad-scale model estimates. The estimates of the intermediate level model (Fig 8A) showed better fit in comparison to the dis-aggregated estimates (Fig 8B) and aggregated estimates (Fig 8C), as unlike the intermediate-scale estimates, the latter (broad-scale and fine-scale model estimates) are not informed by the observed data. In addition, the dis-aggregation of broad-scale model estimates (used in Fig 8B) which we used to show the remaining option in the absence of intermediate level data, is less optimal since it assigns equal probabilities to all geographic units within a single department.

Our fine-scale model resulted in a better fit than the broad-scale model in estimating mobility at the intermediate level, with the latter particularly overestimating mobility (Fig 8B). Further analysis of municipality level flows pertaining to the 147 single-municipalities units in the intermediate level data (Fig 8D) also show reasonably good fit, especially considering that single-municipality units are biased towards large population centers. In addition, analysis of the residuals in all comparisons of model versus observation data revealed that the fine-scale model provides a better fit (based on correlation) comparable to those obtained by fitting the intermediate-level data (Fig 8E–8H).

## Discussion

In this study, we have assessed the factors that drive migration patterns within Colombia and proposed a novel spatial interaction modeling approach to predict migration at a finer spatial scale than the one at which migration data are either recorded or made available. Our spatial interaction model is unconstrained both at the origin and destination sides (i.e. not normalized over the total inflows/outflows to/from each location), and thus could potentially result in over predictions. At the same time, our novel approach provides a much-needed flexibility to potentially predict the total inflows/outflows to/from each location at any scale, as opposed to being constrained by observed total inflows and outflows at a fixed spatial scale[56–58]. This would enable predict migration at a finer scale than the one at which migration data are generally available. Our approach also assumes that observed migration patterns are relatively stable and not influenced by large unobserved events.

Our estimated department level migration flows based on the fine-scale model (i.e., based on estimated municipality level migration flows aggregated to the department level) demonstrated a good agreement with the observed department level migration flows extracted from the 2005 census (Figs 4–6). At the municipality level, our results provided estimated migration flows among the 1,122 municipalities in Colombia with 18% of migrations happening within the same department. Validation of these estimates using observed intermediate level migration flows (based on 276 census units including 147 single municipality units) demonstrated that our estimates are robust and comparable to those that would be obtained by fitting directly to the intermediate level data (rather than holding them out for validation).

Comparison of our model at the department, intermediate, and municipality levels revealed differences between the corresponding broad-scale, intermediate-scale and fine-scale model fits. Distance between origin and destination was confirmed to be the most significant mediating driver of migration, i.e. those that facilitate and consolidate migration [20], while its coefficient values varied significantly across scales (Fig 3). The magnitude of distance’s effect on migration in our fine-scale model is consistent with findings from other countries across the world based on a classic gravity model with comparable population sizes and distances [57]. However, while increasing from broad-scale to intermediate-scale, consistently with those findings from other countries, the effect of distance became lower at the fine-scale. This seems to be due to the larger effects of other factors, including contiguity between origin and destination and the urban/rural status of the latter, with both the broad-scale and intermediate-scale models offsetting such effects by penalizing remote destinations.

At the municipality level, the effect of population size in the best fine-scale model showed large population leading to more emigration and less immigration. This was reinforced by the positive effect on migration of the destination being among the lowest tenth percentile in population. At the same time, our best fine-scale model showed increased migration towards destinations that are in the top 90^th^ percentile of population, which we labeled major population centers, in addition to strong positive and moderate negative effects of urban proportions at the destination and origin, respectively. These results suggest that, except for very few major population centers, population movement is strongly characterized by movement from rural to urban administrative units [59] and weakly by movement from higher to lower populated administrative units; with the latter possibly as a result of model structure trying to offset the effect of other covariates with stronger effects. Our results constitute an important deviation from the basic assumption of the gravity model, which in its original form assumes larger population, both at origins and destinations, leading to larger movements in both directions [43].

Regarding the effect of economic status, our best fine-scale model seems to show that migration in Colombia is driven by economic depression at origins, but not necessarily by economic attractiveness at destinations, suggesting a propensity to migrate as a result of deprivation at origins and perceived economic opportunities at destinations [60]. Van Hear et al. (2018) suggested a categorization of drivers of migration into predisposing, proximate, precipitating, and mediating [20]. Our best fine-scale model includes predisposing drivers such as high and low level of urbanization at the destination and origin, respectively, proximate drivers such as lack of economic opportunities and population pressure at the origin, and mediating drivers such as distance and contiguity between origin and destination.

Our modeling approach is not without limitations. Because aggregated municipality level estimates were compared to observed department level data [60], which do not include intra-department migration, the modeling approach may have poorly captured internal migration within departments which affect about six percent of all routes. The fact that administrative units at different levels are formed arbitrarily, both in terms of geographic size (which affects distances) and population (affecting many of the covariates we used), our models may have biases arising from such effect (also known as Modifiable Area Unit Problem). This problem might have contributed to discrepancies between municipality-level, intermediate-level and department-level estimates and observed data. Note that the intermediate level migration data are made up of single municipalities and groups of contiguous municipalities, which, when comparing results, may create potential biases depending on how each group was formed.

Although migration represents a form of long-term human mobility, it has been widely demonstrated that migration data can serve as reliable proxy for the relative strength of human connectivity across multiple temporal and spatial scales [7,14]. Similarly, it has been suggested that international migration provides added effect of enhancing shorter-term occupational mobility [61]. Human mobility is an important factor in the study of several social, economic, political and biological systems that operate at various temporal and spatial scales. In particular, global health has been under a growing threat due to rapid spread of infectious diseases at continental scales. The expansion of chikungunya since 2013 and the recent invasion by the Zika virus in the Americas, for instance, have caused significant human suffering and economic losses in multiple countries across the region [62,63]. The spread of infectious diseases is often compounded by the multitude of ways infected individuals travel, both locally and internationally, opening opportunities for the disease to spread [44,64]. Coupled with the ecology of several infectious diseases, often characterized by significant heterogeneity at fine spatial scales [15,16], the need for estimating human mobility at fine spatial scales is well recognized. This study represents a first contribution toward addressing the current lack of fine-scale migration and human mobility data in Colombia for supporting the research in the spread of infectious disease and beyond. Furthermore, the spatial interactive modeling approach we used can be applied in many other countries across Africa, Asia and Latin America and the Caribbean, characterized by similar socio-economic conditions.

## Conclusions

Our model indicated that distance and contiguity are the most significant variables driving migration at all scales, while their values could vary depending on the scale being considered. At the same time our results also showed that the coefficients of other variables differ either in their direction (origin and destination population, origin’s urban proportion) or their significance (destination’s per capita gross cell product and the quality of being a major population center) at different scales. The strong influence of distance, contiguity and the quality of being a major population center at the municipality level mean that individuals tend to migrate to nearby municipalities, especially to those destinations with large population size. Given that migration flows can be used as reliable proxy for short term connectivity, those locations with higher migrant flows (either as origin or destination) are also the ones characterized by higher level of mobility of humans. Our model estimates enable the use migration data to infer migration at higher spatial details in locations such as Colombia where these data are lacking, and further enables the use of those estimates to infer human mobility essential for other systems including modeling the spread of infectious diseases.

## Supporting information

S1 Fig Parameter traces in the best fine-scale model all showing convergence. Results of Gelman Rubin convergence diagnostics test also confirmed convergence with potential scale reduction factors equal 1 for all variables.
(PDF)

S2 Fig Predicted versus observed migrants (in log scale) between each pair of Admin-1 units with (A) predictions based on the broad-scale model applied to data at the broad-scale level (B) predictions based on the broad-scale model applied to data at the fine-scale level (C) predictions based on the intermediate-scale model applied to data on the intermediate level (D) predictions based on the intermediate-scale model applied to data on the fine-scale level.
(PDF)

S3 Fig Posterior distribution of parameter in the best broad-scale model all showing convergence. Note that the set of significant covariates is different from that of the fines-scale model shown in Figs 3 and S1.
(PDF)

S1 Table Intermediate level geographic units based on census units after aggregation aiming uniquely identifiable origin and destination locations. The original list of 533 locations was collapsed to 276 such locations, identified by IDs in the second column, which correspond to the original IDs shown in S2 Table.
(PDF)

S2 Table 533 geographic units in Colombia for which IPUMS migration data is available. Note that these units are aggregated into 276 units shown in S1 Table.
(PDF)

S3 Table Coefficients of the best model under the broad-scale modeling approach. (PDF)

S4 Table Coefficients of the best model under the intermediate-scale modeling approach. (PDF)
