# Supplementary material for: Modeling human migration across spatial scales in Colombia
Source: PLoS One. 2020 May 7;15(5):e0232702. doi: 10.1371/journal.pone.0232702 (PMC7205305; doi:10.1371/journal.pone.0232702)
Supplement: S1 Fig — Results of Gelman Rubin convergence diagnostics test also confirmed convergence with potential scale reduction factors equal 1 for all variables. (PDF) [file pone.0232702.s001.pdf]

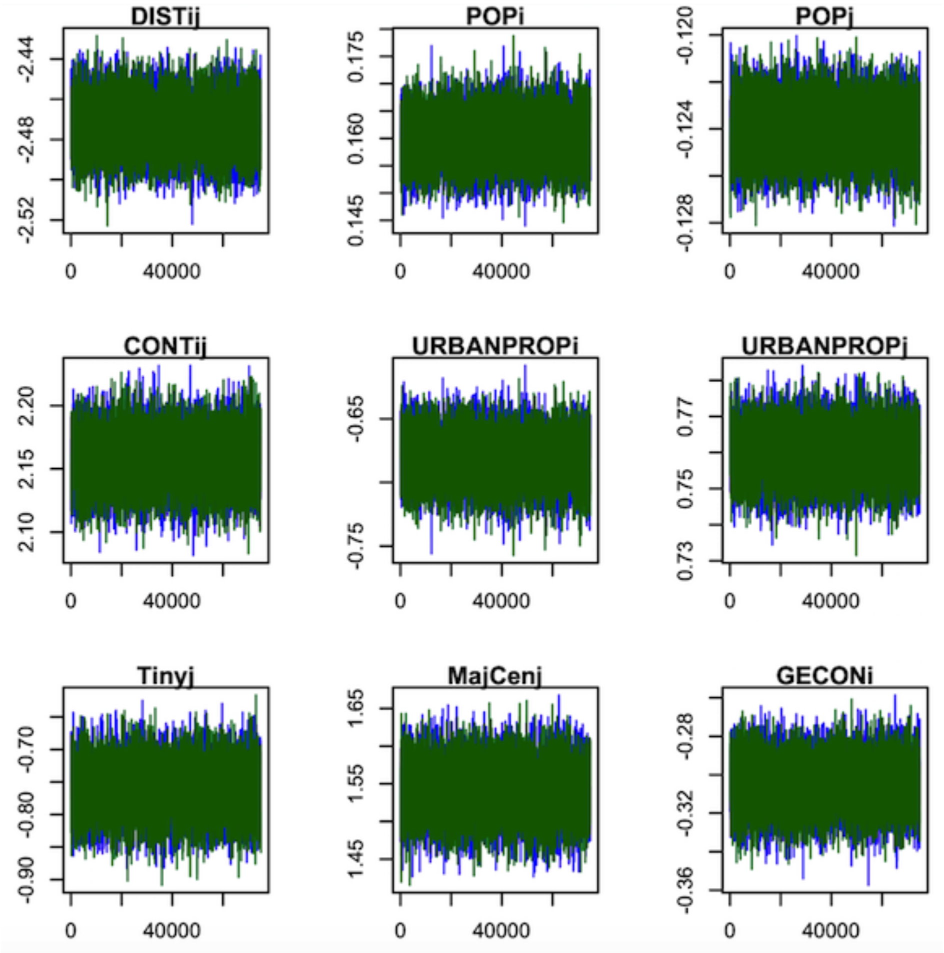

S1 Figure: Parameter traces in the best fine-scale model all showing convergence. Results of Gelman Rubin convergence diagnostics test also confirmed convergence with potential scale reduction factors equal 1 for all variables.
