# Supplementary material for: Modeling human migration across spatial scales in Colombia
Source: PLoS One. 2020 May 7;15(5):e0232702. doi: 10.1371/journal.pone.0232702 (PMC7205305; doi:10.1371/journal.pone.0232702)
Supplement: S2 Fig — Predicted versus observed migrants (in log scale) between each pair of Admin-1 units with (A) predictions based on the broad-scale model applied to data at the broad-scale level (B) predictions based on the broad-scale model applied to data at the fine-scale level (C) predictions based on the intermediate-scale model applied to data on the intermediate level (D) predictions based on the intermediate-scale model applied to data on the fine-scale level. (PDF) [file pone.0232702.s002.pdf]

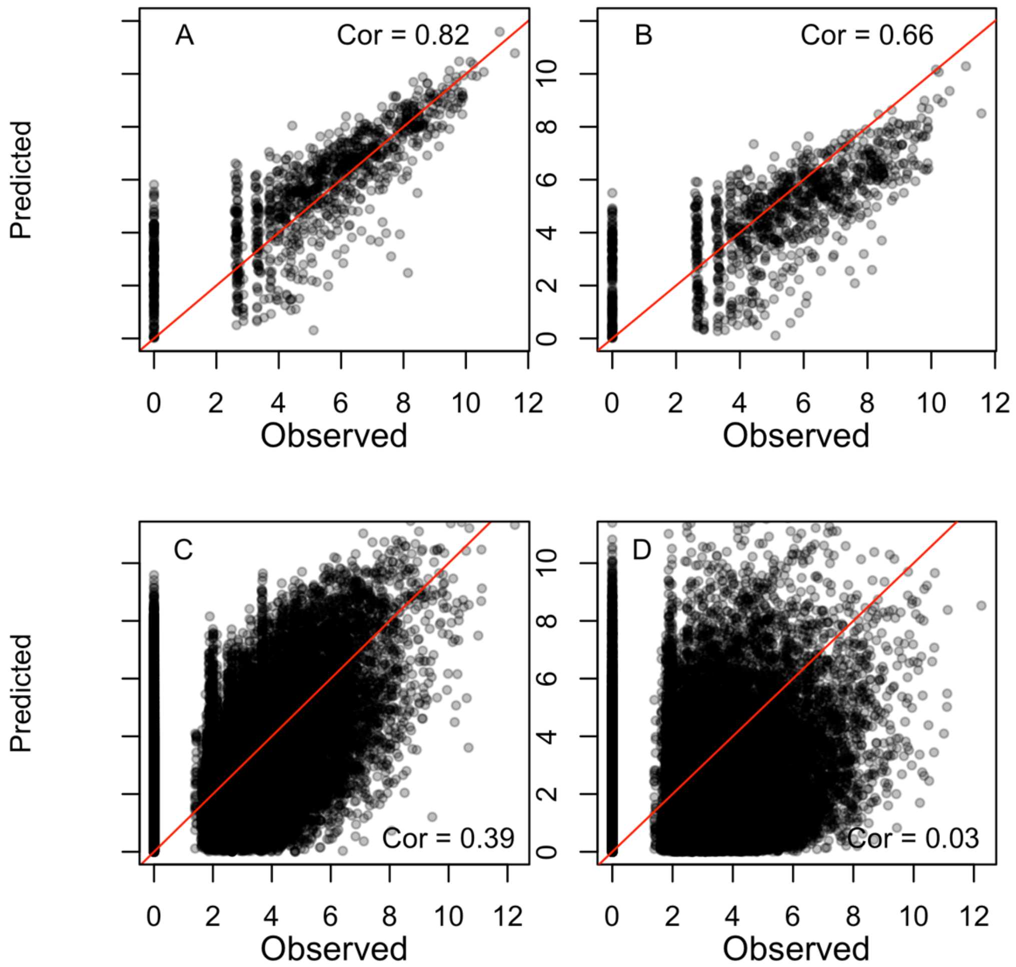

S2 Figure: Predicted versus observed migrants (in log scale) between each pair of Admin-1 units with (A) predictions based on the broad-scale model applied to data at the broad-scale level (B) predictions based on the broad-scale model applied to data at the fine-scale level (C) predictions based on the intermediate-scale model applied to data on the intermediate level (D) predictions based on the intermediate-scale model applied to data on the fine-scale level.
