# Supplementary material for: Modeling human migration across spatial scales in Colombia
Source: PLoS One. 2020 May 7;15(5):e0232702. doi: 10.1371/journal.pone.0232702 (PMC7205305; doi:10.1371/journal.pone.0232702)
Supplement: S3 Fig — Note that the set of significant covariates is different from that of the fines-scale model shown in Figs 3 and S1. (PDF) [file pone.0232702.s003.pdf]

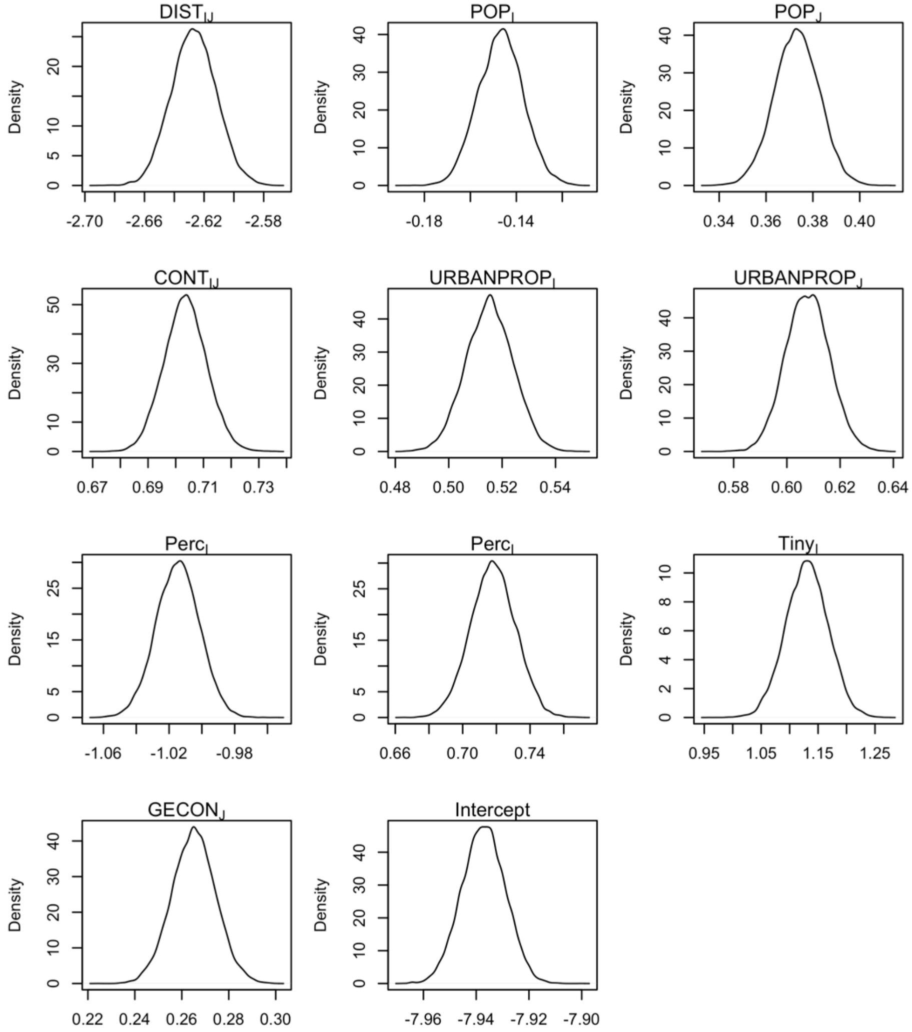

S3 Figure: Posterior distribution of parameter in the best broad-scale model all showing convergence. Note that the set of significant covariates is different from that of the fines-scale model shown in Figure 2 and Figure S1.
