# Supplementary material for: Modeling human migration across spatial scales in Colombia
Source: PLoS One. 2020 May 7;15(5):e0232702. doi: 10.1371/journal.pone.0232702 (PMC7205305; doi:10.1371/journal.pone.0232702)
Supplement: S1 Table — The original list of 533 locations was collapsed to 276 such locations, identified by IDs in the second column, which correspond to the original IDs shown in S2 Table. (PDF) [file pone.0232702.s004.pdf]

S1 Table. Intermediate level geographic units based on census units after aggregation aiming uniquely identifiable origin and destination locations. The original list of 533 locations was collapsed to 276 such locations, identified by IDs in the second column, which correspond to the original IDs shown in S2 Table.

New ID	Includes old IDs
1	19
2	1
3	24
4	41
5	29
6	34
7	8
8	22
9	7
10	26
11	28
12	32
13	66
14	10
15	44
16	16
17	27
18	38
19	3
20	5
21	46
22	31
23	14
24	23
25	39
26	74
27	76
28	73
29	80
30	77
31	75,86
32	79
33	6
34	13,18

35	2,12,40,61

New ID	Includes old IDs
36	88
37	93
38	96
39	105
40	94
41	89
42	95
43	97,99
44	90
45	110
46	100
47	115
48	143
49	117
50	113
51	118
52	114
53	17,21,42,50
54	78,83
55	81,82
56	152
57	154,164
58	150,155
59	151
60	148
61	160
62	157
63	165
64	161
65	163
66	166
67	169
68	183
69	177
70	184
71	191
72	178
73	181

74	186

New ID	Includes old IDs
75	195
76	187
77	188
78	199
79	185
80	175
81	207
82	210
83	206
84	202
85	204
86	208
87	205
88	201
89	215
90	214
91	231
92	230
93	233
94	219
95	228
96	216
97	229
98	223
99	218
100	225
101	227
102	226
103	245
104	270
105	247
106	281
107	235
108	236
109	243
110	242
111	238
112	246

113	239
New ID	Includes old IDs
114	237
115	240
116	249
117	278
118	244
119	279
120	248
121	92,101
122	91,106
123	103,104
124	289
125	293
126	286,292
127	282
128	84,85,87
129	153,159,162
130	149,156,158
131	190,192,193
132	180,198
133	212,213
134	203,209
135	211
136	294
137	299
138	295
139	300,301
140	296
141	316
142	313
143	318
144	312
145	317
146	315
147	232
148	220,221,234
149	222
150	319
151	321

New ID	Includes old IDs
152	323
153	332
154	333
155	334
156	269
157	361
158	346
159	353
160	350
161	369
162	363
163	349
164	107,108,109
165	98,102,111
166	385
167	392
168	380
169	283,288
170	379
171	383
172	378
173	376
174	382
175	30,33,35,37,45,47,55,56,57,58,59,60,63,64,65,72
176	179,194
177	176,182,200
178	401
179	396
180	400
181	399
182	398
183	402
184	410
185	404
186	412
187	411
188	414
189	419
190	415

New ID	Includes old IDs
191	413
192	217,224
193	451
194	455
195	449
196	446
197	444
198	452
199	442
200	450
201	457,469
202	462
203	461
204	459
205	471
206	473
207	464
208	456
209	460
210	119,120,121,122,123,124,125,127,128
211	4,9,15,20,25,36,43,48,49,51,52,53,54,62,67,68,69,70,71
212	487
213	499
214	484
215	497
216	495
217	491
218	492
219	485
220	503,504
221	488
222	500
223	490
224	502
225	505
226	501
227	508
228	189,196,197

New ID	Includes old IDs
229	514
230	285,287,290,291
231	517
232	516
233	524
234	523
235	522
236	526
237	528
238	167,168,170,171,172,173,174
239	322
240	324,325
241	328
242	530
243	337,342
244	336,341
245	348,367
246	352,355,362
247	307,309
248	395,397
249	405,407
250	311,314
251	417,424
252	494,496
253	493,511
254	489,509,510
255	364,365,366,368
256	512,513,515
257	377,384,386,387
258	521,525,527
259	403,406,408,409
260	335,338,339,340,343
261	445,453,454
262	112,126,129,130,131,132,133,134,135,136,137,138,139,140,141,142,144,145,146,147
263	381,388,389,390,391,393,394
264	370,371,373,374,375
265	529,531,532,533
266	468,472,482,483
267	458,463,470,474,475,480,481

New ID	Includes old IDs
268	486,498,506,507
269	320,326,327,329,330,331
270	518,519,520
271	443,447,448
272	344,345,347,351,354,356,357,358,359,360,372
273	465,466,467,476,477,478,479
274	297,298,302,303,304,305,306,308,310
275	241,250,251,252,253,254,255,256,257,258,259,260,261,262, 263,264,265,266,267,268,271,272,273,274,275,276,277,280
276	416,418,420,421,422,423,425,426,427,428,429, 430,431,432,433,434,435,436,437,438,439,440,441
