# Supplementary material for: Modeling human migration across spatial scales in Colombia
Source: PLoS One. 2020 May 7;15(5):e0232702. doi: 10.1371/journal.pone.0232702 (PMC7205305; doi:10.1371/journal.pone.0232702)
Supplement: S2 Table — Note that these units are aggregated into 276 units shown in S1 Table. (PDF) [file pone.0232702.s005.pdf]

**S2 Table. 533 Geographic units in Colombia for which IPUMS migration data is available. Note that these units are aggregated into 276 units shown in S1 Table.**

| Department | ID | Admin Name |
|---|---|---|
| ANTIOQUIA | 1 | Medellín |
| ANTIOQUIA | 2 | Abejorral, La Unión |
| ANTIOQUIA | 3 | Amagá |
| ANTIOQUIA | 4 | Andes |
| ANTIOQUIA | 5 | Apartado |
| ANTIOQUIA | 6 | Arboletes, San Juan de Uraba |
| ANTIOQUIA | 7 | Barbosa |
| ANTIOQUIA | 8 | Bello |
| ANTIOQUIA | 9 | Betania, Cuidad Bolivar |
| ANTIOQUIA | 10 | Caldas |
| ANTIOQUIA | 11 | Carepa |
| ANTIOQUIA | 12 | Carmen de Viboral |
| ANTIOQUIA | 13 | Caucasia |
| ANTIOQUIA | 14 | Chigorodo |
| ANTIOQUIA | 15 | Concordia |
| ANTIOQUIA | 16 | Copacabana |
| ANTIOQUIA | 17 | Dabeiba, Mutata |
| ANTIOQUIA | 18 | El Bagre, Nechi |
| ANTIOQUIA | 19 | Envigado |
| ANTIOQUIA | 20 | Fredonia |
| ANTIOQUIA | 21 | Frontino, Murindo, Vigia del Fuerte |
| ANTIOQUIA | 22 | Girardota |
| ANTIOQUIA | 23 | Guarne |
| ANTIOQUIA | 24 | Itaguí |
| ANTIOQUIA | 25 | Ituango |
| ANTIOQUIA | 26 | La Ceja |
| ANTIOQUIA | 27 | La Estrella |
| ANTIOQUIA | 28 | Marinilla |
| ANTIOQUIA | 29 | Necocli |
| ANTIOQUIA | 30 | Puerto Berrio |
| ANTIOQUIA | 31 | Rionegro |
| ANTIOQUIA | 32 | Sabaneta |
| ANTIOQUIA | 33 | San Carlos |
| ANTIOQUIA | 34 | San Pedro de Uraba |
| ANTIOQUIA | 35 | San Vicente |
| ANTIOQUIA | 36 | Santa Bárbara, La Pintada |

| Department | ID | Admin Name |
| --- | --- | --- |
| ANTIOQUIA | 37 | Santa Rosa de Osos |
| ANTIOQUIA | 38 | Santuario |
| ANTIOQUIA | 39 | Segovia |
| ANTIOQUIA | 40 | Sonsón |
| ANTIOQUIA | 41 | Turbo |
| ANTIOQUIA | 42 | Urrao |
| ANTIOQUIA | 43 | Yarumal |
| ANTIOQUIA | 44 | Zaragoza |
| ANTIOQUIA | 45 | Cáceres, Anorí |
| ANTIOQUIA | 46 | Tarazá, Valdivia |
| ANTIOQUIA | 47 | Angostura, Campamento |
| ANTIOQUIA | 48 | Briceño, San Andres, Toledo |
| ANTIOQUIA | 49 | Buriticá, Peque, Sabanalarga |
| ANTIOQUIA | 50 | Abriaquí, Cañasgordas, Uramita |
| ANTIOQUIA | 51 | Santafe de Antioquia, Giraldo, Caicedo |
| ANTIOQUIA | 52 | Belmira, San José de la Montaña, Liborina, Olaya |
| ANTIOQUIA | 53 | Ebejico, San Jerónimo, Sopetrán |
| ANTIOQUIA | 54 | Anza, Betulia |
| ANTIOQUIA | 55 | Amalfi, Remedios, Yondó |
| ANTIOQUIA | 56 | Vegachí, Yali, Yolombó |
| ANTIOQUIA | 57 | Carolina, Gomez Plata, Guadalupe |
| ANTIOQUIA | 58 | Caracolí, Maceo, San Roque |
| ANTIOQUIA | 59 | Puerto Naré (La Magdalena), Puerto Triunfo |
| ANTIOQUIA | 60 | San Francisco, San Luis |
| ANTIOQUIA | 61 | Argelia, Nariño |
| ANTIOQUIA | 62 | Cisneros, Santo Domingo, Hispania, Pueblorrico, Salgar |
| ANTIOQUIA | 63 | Alejandría, Concepción, San Rafael |
| ANTIOQUIA | 64 | Guatape, Peñol |
| ANTIOQUIA | 65 | Cocorna, Granada |
| ANTIOQUIA | 66 | Retiro, Montebello |
| ANTIOQUIA | 67 | Caramanta, Valparaíso |
| ANTIOQUIA | 68 | Jardín, Támesis |
| ANTIOQUIA | 69 | Jericó, Tarso |
| ANTIOQUIA | 70 | Titiribí, Venecia |
| ANTIOQUIA | 71 | Armenia, Heliconia, Angelópolis |
| ANTIOQUIA | 72 | Don Matías, Entrerrios, San Pedro |
| ATLÁNTICO | 73 | Barranquilla |
| ATLÁNTICO | 74 | Baranoa |
| ATLÁNTICO | 75 | Campo de la Cruz |

| Department | ID | Admin Name |
|---|---|---|
| ATLÁNTICO | 76 | Malambo |
| ATLÁNTICO | 77 | Puerto Colombia |
| ATLÁNTICO | 78 | Sabanalarga |
| ATLÁNTICO | 79 | Santo Tomas |
| ATLÁNTICO | 80 | Soledad |
| ATLÁNTICO | 81 | Galapa, Tubará |
| ATLÁNTICO | 82 | Usiacurí, Juan de Acosta, Piojo |
| ATLÁNTICO | 83 | Polonuevo, Sabanagrande |
| ATLÁNTICO | 84 | Palmar de Varela, Ponedera |
| ATLÁNTICO | 85 | Candelaria, Manatí |
| ATLÁNTICO | 86 | Santa Lucía, Suán |
| ATLÁNTICO | 87 | Luruaco, Repelón |
| BOGOTÁ | 88 | Bogotá D. C. |
| BOLÍVAR | 89 | Cartagena |
| BOLÍVAR | 90 | Achí, Montecristo, San Jacinto del Cauca |
| BOLÍVAR | 91 | Arjona |
| BOLÍVAR | 92 | Calamar, El Guamo |
| BOLÍVAR | 93 | El Carmen de Bolívar |
| BOLÍVAR | 94 | Magangué |
| BOLÍVAR | 95 | María La Baja |
| BOLÍVAR | 96 | Mompós |
| BOLÍVAR | 97 | Morales, Arenal |
| BOLÍVAR | 98 | Pinillos, Tiquisio |
| BOLÍVAR | 99 | Rio Viejo, Regidor |
| BOLÍVAR | 100 | San Jacinto |
| BOLÍVAR | 101 | San Juan Nepomuceno |
| BOLÍVAR | 102 | San Martín de Loba, El Peñón, Hatillo de Loba |
| BOLÍVAR | 103 | San Pablo, Cantagallo, Simití |
| BOLÍVAR | 104 | Santa Rosa del Sur |
| BOLÍVAR | 105 | Talaiga Nuevo, Circuco |
| BOLÍVAR | 106 | Turbaco, Turbana |
| BOLÍVAR | 107 | Santa Catalina, Clemencia, Santa Rosa |
| BOLÍVAR | 108 | San Estanislao, Villanueva |
| BOLÍVAR | 109 | Mahates, Arroyohondo, Soplaviento, San Cristóbal |
| BOLÍVAR | 110 | Córdoba, Zambrano |
| BOLÍVAR | 111 | Barranco de Loba, Altos de Rosario, Margarita, San Fernando |
| BOYACÁ | 112 | Tunja |
| BOYACÁ | 113 | Chiquinquirá |
| BOYACÁ | 114 | Duitama |

| Department | ID | Admin Name |
|---|---|---|
| BOYACÁ | 115 | Moniquirá |
| BOYACÁ | 116 | Paipa |
| BOYACÁ | 117 | Puerto Boyacá |
| BOYACÁ | 118 | Sogamoso |
| BOYACÁ | 119 | Chiscas, Cubará, Guicán |
| BOYACÁ | 120 | El Cocuy, El Espino, Guacamayas, Panqueba, San Mateo |
| BOYACÁ | 121 | Covarachía, Soatá, Tipacoque |
| BOYACÁ | 122 | Boavita, La Uvita, Jericó |
| BOYACÁ | 123 | Chita, Socota |
| BOYACÁ | 124 | Sativanorte, Sativasur, Susacón, Belén, Tutaza |
| BOYACÁ | 125 | Paz De Río, Socha, Tasco |
| BOYACÁ | 126 | Aquitania, Labranzagrande, Pajarito, Paya, Pisba |
| BOYACÁ | 127 | Busbanza, Gámeza, Mongua, Monguí, Tópaga, Corrales |
| BOYACÁ | 128 | Beteitiva, Cerinza, Floresta, Santa Rosa de Viterbo |
| BOYACÁ | 129 | Cuitiva, Firavitoba, Iza, Nobsa, Tibasosa |
| BOYACÁ | 130 | Pesca, Tota, Zetaquirá |
| BOYACÁ | 131 | Cómbita, Oicatá, Sotaquirá |
| BOYACÁ | 132 | Chivatá, Toca, Tuta |
| BOYACÁ | 133 | Siachoque, Soracá, Ciénega, Rondón, Viracachá |
| BOYACÁ | 134 | Berbeo, Campohermoso, Miraflores, Páez, San Eduardo |
| BOYACÁ | 135 | Chivor, Guayatá, San Luis de Gaceno, Santa María |
| BOYACÁ | 136 | Almeida, Garagoa, Macanal |
| BOYACÁ | 137 | Guateque, Somondoco, Sutatenza, Tenza |
| BOYACÁ | 138 | Chinavita, La Capilla, Pachavita, Umbita |
| BOYACÁ | 139 | Boyacá, Nuevo Colón, Jenesano, Tibaná, Ramiriquí |
| BOYACÁ | 140 | Ventaquemada, Turmequé |
| BOYACÁ | 141 | Cucaita, Chiquiza, Motavita, Samacá, Sora, Ráquira |
| BOYACÁ | 142 | Arcabuco, Gachantivá, Villa de Leyva, Sáchica, Santa Sofía |
| BOYACÁ | 143 | Chitaraque, San José De Pare, Santana, Togüí |
| BOYACÁ | 144 | Saboyá, Sacamá, San Miguel de Sema, Sutamarchán, Tinjacá |
| BOYACÁ | 145 | Buenavista, Caldas, Coper, Maripí, Muzo |
| BOYACÁ | 146 | Briceño, Pauna, San Pablo de Borbur, Tununguá |
| BOYACÁ | 147 | La Victoria, Otanche, Quípama |
| CALDAS | 148 | Manizales |
| CALDAS | 149 | Aguadas |
| CALDAS | 150 | Anserma |
| CALDAS | 151 | Chinchiná |
| CALDAS | 152 | La Dorada |
| CALDAS | 153 | Manzanares |

| Department | ID | Admin Name |
|---|---|---|
| CALDAS | 154 | Neira |
| CALDAS | 155 | Palestina |
| CALDAS | 156 | Pensilvania |
| CALDAS | 157 | Riosucio |
| CALDAS | 158 | Marulanda, Salamina |
| CALDAS | 159 | Samaná, Norcasia |
| CALDAS | 160 | Marmato, Supía |
| CALDAS | 161 | Villamaría |
| CALDAS | 162 | Marquetalia, Victoria |
| CALDAS | 163 | La Merced, Pácora |
| CALDAS | 164 | Filadelfia, Aranzazu |
| CALDAS | 165 | Belalcázar, Risaralda, San José, Viterbo |
| CAQUETÁ | 166 | Florencia |
| CAQUETÁ | 167 | Cartagena del Chairá, El Doncello |
| CAQUETÁ | 168 | Puerto Rico |
| CAQUETÁ | 169 | San Vicente del Caguán |
| CAQUETÁ | 170 | Valparaiso, Solita |
| CAQUETÁ | 171 | Currillo, San José de la Fragua |
| CAQUETÁ | 172 | Albania, Belén de los Andaquies, Morelia |
| CAQUETÁ | 173 | Solano, Milán |
| CAQUETÁ | 174 | El Paujíl, Getucha, La Montañita |
| CAUCA | 175 | Popayán |
| CAUCA | 176 | Bolívar, Sucre |
| CAUCA | 177 | Cajibío |
| CAUCA | 178 | Caldono |
| CAUCA | 179 | Padilla, Caloto |
| CAUCA | 180 | El Tambo |
| CAUCA | 181 | Guapi |
| CAUCA | 182 | Almaguer, La Vega |
| CAUCA | 183 | Morales |
| CAUCA | 184 | Páez |
| CAUCA | 185 | Patía |
| CAUCA | 186 | Piendamó |
| CAUCA | 187 | Puerto Tejada |
| CAUCA | 188 | Santander de Quilichao, Villa Rica |
| CAUCA | 189 | Silvia |
| CAUCA | 190 | Suárez |
| CAUCA | 191 | Timbío |
| CAUCA | 192 | Timbiquí |

| Department | ID | Admin Name |
|---|---|---|
| CAUCA | 193 | López, Buenos Aires |
| CAUCA | 194 | Corinto, Miranda |
| CAUCA | 195 | Toribío, Jambaló |
| CAUCA | 196 | Puracé, Inzá, Totoró |
| CAUCA | 197 | La Sierra, Rosas, Sotará |
| CAUCA | 198 | Argelia, Balboa |
| CAUCA | 199 | Florencia, Mercaderes |
| CAUCA | 200 | San Sebastián, Santa Rosa, Piamonte |
| CESAR | 201 | Valledupar, Pueblo Bello |
| CESAR | 202 | Aguachica |
| CESAR | 203 | Agustín Codazzi |
| CESAR | 204 | Bosconia |
| CESAR | 205 | Chimichagua |
| CESAR | 206 | Chiriguaná |
| CESAR | 207 | Curumaní |
| CESAR | 208 | El Copey |
| CESAR | 209 | Becerril, La Jagua de Ibirico |
| CESAR | 210 | Manaure, La Paz, San Diego |
| CESAR | 211 | Astrea, El Paso |
| CESAR | 212 | Pailitas, Tamalameque |
| CESAR | 213 | Gamarra, La Gloria, Pelaya |
| CESAR | 214 | González, Río de Oro |
| CESAR | 215 | San Alberto, San Martín |
| CÓRDOBA | 216 | Montería |
| CÓRDOBA | 217 | Ayapel |
| CÓRDOBA | 218 | Cereté |
| CÓRDOBA | 219 | Chinú |
| CÓRDOBA | 220 | Cienaga de Oro |
| CÓRDOBA | 221 | Lorica, Cotorra |
| CÓRDOBA | 222 | Montelíbano, La Apartada, Puerto Libertador |
| CÓRDOBA | 223 | Planeta Rica |
| CÓRDOBA | 224 | Buenavista, Pueblo Nuevo, San Carlos |
| CÓRDOBA | 225 | Sahagún |
| CÓRDOBA | 226 | San Andrés de Sotavento |
| CÓRDOBA | 227 | San Bernardo del Viento |
| CÓRDOBA | 228 | San Pelayo |
| CÓRDOBA | 229 | Tierralta |
| CÓRDOBA | 230 | Valencia |
| CÓRDOBA | 231 | Canalete, Los Córdobas |

| Department | ID | Admin Name |
|---|---|---|
| CÓRDOBA | 232 | Moñitos, Puerto Escondido |
| CÓRDOBA | 233 | Purísima, San Antero |
| CÓRDOBA | 234 | Chimá, Momil |
| CUNDINAMARCA | 235 | Cajicá |
| CUNDINAMARCA | 236 | Chía |
| CUNDINAMARCA | 237 | Facatativá |
| CUNDINAMARCA | 238 | Funza |
| CUNDINAMARCA | 239 | Fusagasugá |
| CUNDINAMARCA | 240 | Girardot |
| CUNDINAMARCA | 241 | Guaduas |
| CUNDINAMARCA | 242 | Madrid |
| CUNDINAMARCA | 243 | Mosquera |
| CUNDINAMARCA | 244 | Pacho |
| CUNDINAMARCA | 245 | Sibate |
| CUNDINAMARCA | 246 | Soacha, Granada |
| CUNDINAMARCA | 247 | Ubaté |
| CUNDINAMARCA | 248 | Villeta |
| CUNDINAMARCA | 249 | Zipaquirá |
| CUNDINAMARCA | 250 | Yacopí, Puerto Salgar |
| CUNDINAMARCA | 251 | Caparrapí, Utica |
| CUNDINAMARCA | 252 | La Palma, La Peña |
| CUNDINAMARCA | 253 | El Peñón, Paime, San Cayetano, Topaipí, Villagomez |
| CUNDINAMARCA | 254 | Carmen de Carupa, Simijaca, Susa |
| CUNDINAMARCA | 255 | Fúquene, Guachetá, Lenguazaque |
| CUNDINAMARCA | 256 | Manta, Tibirita, Villapinzón |
| CUNDINAMARCA | 257 | Chocontá, Machetá |
| CUNDINAMARCA | 258 | Suesca, Cucunubá, Sutatausa |
| CUNDINAMARCA | 259 | Cogua, Nemocón, Tausa |
| CUNDINAMARCA | 260 | Guatavita, Sesquilé, Gachancipá |
| CUNDINAMARCA | 261 | Choachí, Fómeque |
| CUNDINAMARCA | 262 | Gachetá, Gama, Junín |
| CUNDINAMARCA | 263 | Guasca, La Calera |
| CUNDINAMARCA | 264 | Sopó, Tocancipá |
| CUNDINAMARCA | 265 | Caqueza, Chipaque, Ubaque |
| CUNDINAMARCA | 266 | Fosca, Guayabetal, Quetame, Une, Gutiérrez |
| CUNDINAMARCA | 267 | Nimaima, Quebradanegra, Vergara |
| CUNDINAMARCA | 268 | La Vega, San Francisco, Supatá |
| CUNDINAMARCA | 269 | Tabio, Subachoque, El Rosal |
| CUNDINAMARCA | 270 | Cota, Tenjo |

| Department | ID | Admin Name |
| --- | --- | --- |
| CUNDINAMARCA | 271 | Guayabal de Síquima, Albán, Sasaima, Nocaima |
| CUNDINAMARCA | 272 | Bituima, Chaguaní, Quipile, Vianí |
| CUNDINAMARCA | 273 | Beltrán, Guataquí, Pulí, Jerusalén, San Juan de Rio Seco, Anapoima, La Mesa, Nariño |
| CUNDINAMARCA | 274 | Anolaima, Cachipay |
| CUNDINAMARCA | 275 | Bojacá, Zipacón, Tena, San Antonio del Tequendama |
| CUNDINAMARCA | 276 | Tocaima, Apulo |
| CUNDINAMARCA | 277 | Silvania, Tibacuy, Viotá, El Colegio |
| CUNDINAMARCA | 278 | Arbeláez, Pasca |
| CUNDINAMARCA | 279 | Cabrera, Venecia, San Bernardo, Pandi |
| CUNDINAMARCA | 280 | Agua de Dios, Ricaurte, Nilo |
| CUNDINAMARCA | 281 | Gachalá, Paratebueno, Ubalá, Medina |
| CHOCÓ | 282 | Quibdó, Atrato, Medio Atrato, Río Quito |
| CHOCÓ | 283 | Bajo Baudó, Medio Baudó |
| CHOCÓ | 285 | Itsmina, El Cantón del San Pablo, Medio San Juan, Unión Panamericana |
| CHOCÓ | 286 | Riosucio, Carmen del Darien, Belén de Bajirá |
| CHOCÓ | 287 | Tadó, Cértegui, Río Iro |
| CHOCÓ | 288 | Nuquí, Alto Baudó |
| CHOCÓ | 289 | Bagadó, El Carmen de Atrato, Lloró |
| CHOCÓ | 290 | Condoto, Novita |
| CHOCÓ | 291 | El Litoral del San Juan, San José del Palmar, Sipí |
| CHOCÓ | 292 | Acandí, Unguía |
| CHOCÓ | 293 | Bahía Solano, Bojayá, Juradó |
| HUILA | 294 | Neiva |
| HUILA | 295 | Campoalegre |
| HUILA | 296 | Garzón |
| HUILA | 297 | La Plata |
| HUILA | 298 | Pitalito |
| HUILA | 299 | San Agustín |
| HUILA | 300 | Baraya, Colombia, Villavieja |
| HUILA | 301 | Aipe, Tello |
| HUILA | 302 | Palermo, Santa María |
| HUILA | 303 | Algeciras, Rivera |
| HUILA | 304 | Hobo, Iquira, Yaguará, Teruel |
| HUILA | 305 | Gigante, Nataga, Tesalia |
| HUILA | 306 | Agrado, Altamira, Pital, Paicol |
| HUILA | 307 | La Argentina, Tarqui |
| HUILA | 308 | Suaza, Timaná, Guadalupe |
| HUILA | 309 | Elías, Isnos, Oporapa, Saladoblanco |

| Department | ID | Admin Name |
|---|---|---|
| HUILA | 310 | Acevedo, Palestina |
| LA GUAJIRA | 311 | Riohacha, Dibulla |
| LA GUAJIRA | 312 | Barrancas, Hatonuevo |
| LA GUAJIRA | 313 | Fonseca, Distracción |
| LA GUAJIRA | 314 | Maicao, Albania |
| LA GUAJIRA | 315 | Manaure |
| LA GUAJIRA | 316 | San Juan del Cesar |
| LA GUAJIRA | 317 | Uribia |
| LA GUAJIRA | 318 | El Molino, Urumita, La Jagua del Pilar, Villanueva |
| MAGDALENA | 319 | Santa Marta |
| MAGDALENA | 320 | Aracataca, El Retén |
| MAGDALENA | 321 | Ariguaní |
| MAGDALENA | 322 | Ciénaga, Zona Bananera |
| MAGDALENA | 323 | El Banco |
| MAGDALENA | 324 | Fundación, Algarrobo |
| MAGDALENA | 325 | Pivijay |
| MAGDALENA | 326 | Plato, Nueva Granada, Sabanas de San Angel, Santa Bárbara de Pinto |
| MAGDALENA | 327 | San Zenón, Santa Ana, Pijiño del Carmen |
| MAGDALENA | 328 | Puebloviejo, Sitionuevo |
| MAGDALENA | 329 | El Piñón, Zapayán, Remolino, Salamina |
| MAGDALENA | 330 | Cerro San Antonio, Pedraza, Concordia |
| MAGDALENA | 331 | Chivolo, Tenerife |
| MAGDALENA | 332 | Guamal, San Sebastián de Buenavista |
| META | 333 | Villavicencio |
| META | 334 | Acacias |
| META | 335 | Granada |
| META | 336 | Puerto López |
| META | 337 | Mesetas, La Macarena, La Uribe |
| META | 338 | Puerto Rico, Vista Hermosa |
| META | 339 | Mapiripán, Puerto Concordia, Puerto Lleras |
| META | 340 | Puerto Gaitán, San Martín |
| META | 341 | Barranca de Upia, Cabuyaro, Cumaral, El Calvario, Restrepo, San Juanito |
| META | 342 | Fuente de Oro, Lejanías, San Juan de Arama |
| META | 343 | Castilla la Nueva, San Luis de Cubarral, El Castillo, El Dorado, Guamal, San Carlos de Guaroa |
| NARINO | 344 | Pasto, Nariño |
| NARINO | 345 | Barbacoas |
| NARINO | 346 | Cumbal |
| NARINO | 347 | El Tambo, El Peñol |

| Department | ID | Admin Name |
|---|---|---|
| NARINO | 348 | Guaitarilla |
| NARINO | 349 | Ipiales |
| NARINO | 350 | La Unión |
| NARINO | 351 | La Tola, Olaya Herrera |
| NARINO | 352 | Samaniego |
| NARINO | 353 | Sandoná |
| NARINO | 354 | Tumaco |
| NARINO | 355 | Túquerres |
| NARINO | 356 | Mosquera, Maguí, Francisco Pizarro, Roberto Payán |
| NARINO | 357 | El Charco, Santa Bárbara |
| NARINO | 358 | El Rosario, Leiva |
| NARINO | 359 | Cumbitara, Policarpa, Los Andes |
| NARINO | 360 | La Llanada, Linares |
| NARINO | 361 | Mallama, Ricaurte |
| NARINO | 362 | Sapuyés, Providencia, Santacruz |
| NARINO | 363 | Aldana, Guachucal, Cuaspud |
| NARINO | 364 | Contadero, Pupiales, Gualmatán |
| NARINO | 365 | Iles, Ospina, Imués |
| NARINO | 366 | Tangua, Yacuanquer |
| NARINO | 367 | Ancuyá, La Florida, Consaca |
| NARINO | 368 | Funes, Puerres |
| NARINO | 369 | Potosí, Córdoba |
| NARINO | 370 | Arboleda, Buesaco |
| NARINO | 371 | San Lorenzo, San Pedro de Cartago |
| NARINO | 372 | Chachag_í, Taminango |
| NARINO | 373 | Belén, Colón, San Bernardo |
| NARINO | 374 | La Cruz, San Pablo |
| NARINO | 375 | Albán, El Tablón |
| NORTE SANTANDER | 376 | Cúcuta, Puerto Santander |
| NORTE SANTANDER | 377 | Abrego, La Esperanza |
| NORTE SANTANDER | 378 | Los Patios |
| NORTE SANTANDER | 379 | Ocaña |
| NORTE SANTANDER | 380 | Pamplona |
| NORTE SANTANDER | 381 | Sardinata |
| NORTE SANTANDER | 382 | Tibú |
| NORTE SANTANDER | 383 | Villa del Rosario |
| NORTE SANTANDER | 384 | El Tarra, Teorama |
| NORTE SANTANDER | 385 | Convención, El Carmen |
| NORTE SANTANDER | 386 | Hacarí, San Calixto, La Playa |

| Department | ID | Admin Name |
|---|---|---|
| NORTE SANTANDER | 387 | Cáchira |
| NORTE SANTANDER | 388 | Bucarasica, Gramalote, Lourdes, Villa Caro |
| NORTE SANTANDER | 389 | Arboledas, Salazar |
| NORTE SANTANDER | 390 | El Zulia, San Cayetano, Santiago |
| NORTE SANTANDER | 391 | Cucutilla, Pamplonita, Bochalema, Durania |
| NORTE SANTANDER | 392 | Chinácota, Herrán, Ragonvalia |
| NORTE SANTANDER | 393 | Labateca, Toledo |
| NORTE SANTANDER | 394 | Cácota, Chitagá, Mutiscua, Silos |
| QUINDIO | 395 | Armenia |
| QUINDIO | 396 | Calarcá |
| QUINDIO | 397 | Circasia, Salento |
| QUINDIO | 398 | La Tebaida |
| QUINDIO | 399 | Montenegro |
| QUINDIO | 400 | Filandia, Quimbaya |
| QUINDIO | 401 | Buenavista, Génova, Pijao, Córdoba |
| RISARALDA | 402 | Pereira |
| RISARALDA | 403 | Belén de Umbria |
| RISARALDA | 404 | Dosquebradas |
| RISARALDA | 405 | La Virginia, Marsella |
| RISARALDA | 406 | Quinchía |
| RISARALDA | 407 | Santa Rosa de Cabal |
| RISARALDA | 408 | Guatica, Mistrato |
| RISARALDA | 409 | Apía, Pueblo Rico |
| RISARALDA | 410 | Balboa, Santuario, La Celia |
| SANTANDER | 411 | Bucaramanga |
| SANTANDER | 412 | Barrancabermeja |
| SANTANDER | 413 | Cimitarra |
| SANTANDER | 414 | Floridablanca |
| SANTANDER | 415 | Girón |
| SANTANDER | 416 | Piedecuesta |
| SANTANDER | 417 | Puerto Wilches |
| SANTANDER | 418 | Rionegro |
| SANTANDER | 419 | San Gil |
| SANTANDER | 420 | San Vicente de Chucurí |
| SANTANDER | 421 | Socorro |
| SANTANDER | 422 | Vélez |
| SANTANDER | 423 | El Playón, Matanza, Suratá |
| SANTANDER | 424 | Sabana de Torres, Lebrija |
| SANTANDER | 425 | California, Santa Bárbara, Guaca, Cerrito, Vetas, Tona, Charta |

| Department | ID | Admin Name |
|---|---|---|
| SANTANDER | 426 | Betulia, Los Santos, Zapatoca |
| SANTANDER | 427 | Capitanejo, Carcasi, Concepción, Macaravita |
| SANTANDER | 428 | Enciso, Molagavita, San José de Miranda, San Miguel |
| SANTANDER | 429 | Villanueva, Aratoca, Cepitá, Jordán, Curití |
| SANTANDER | 430 | Mogotes, Onzaga, San Joaquín |
| SANTANDER | 431 | Barichara, Pinchote, Paramo, Palmar, Hato, Galán, Valle de San José, Ocamonte, Cabrera |
| SANTANDER | 432 | Coromoro, Charalá, Encino, Gambita |
| SANTANDER | 433 | El Carmen de Chucuri, Puerto Parra, Simacota |
| SANTANDER | 434 | Aguada, Chipatá, La Paz, Santa Helena del Opón, San Benito |
| SANTANDER | 435 | Chimá, Confines, Contratación, Guadalupe, Palmas del Socorro, El Guacamayo |
| SANTANDER | 436 | Guapota, Oiba, Suaita |
| SANTANDER | 437 | Bolívar, Landazuri |
| SANTANDER | 438 | Barbosa, Güepsa |
| SANTANDER | 439 | Albania, Guavata, Jesus María, Puente Nacional |
| SANTANDER | 440 | Málaga, San Andres |
| SANTANDER | 441 | El Peñón, La Belleza, Sucre, Florián |
| SUCRE | 442 | Sincelejo |
| SUCRE | 443 | Corozal, El Roble |
| SUCRE | 444 | Guaranda, Majagual |
| SUCRE | 445 | Chalán, Ovejas |
| SUCRE | 446 | Sampués |
| SUCRE | 447 | Galeras, Caimito, San Benito Abad |
| SUCRE | 448 | La Unión, San Marcos |
| SUCRE | 449 | San Onofre |
| SUCRE | 450 | San Luis de Sincé |
| SUCRE | 451 | Sucre |
| SUCRE | 452 | Palmito, Tolú, Coveñas |
| SUCRE | 453 | Toluviejo, Coloso |
| SUCRE | 454 | Los Palmitos, Morroa, San Juan de Betulia |
| SUCRE | 455 | Buenavista, San Pedro |
| TOLIMA | 456 | Ibagué |
| TOLIMA | 457 | Ataco |
| TOLIMA | 458 | Chaparral |
| TOLIMA | 459 | Coyaima |
| TOLIMA | 460 | Espinal |
| TOLIMA | 461 | Flandes |
| TOLIMA | 462 | Fresno |
| TOLIMA | 463 | Guamo |

| Department | ID | Admin Name |
|---|---|---|
| TOLIMA | 464 | Honda |
| TOLIMA | 465 | Lérida, Venadillo |
| TOLIMA | 466 | Líbano |
| TOLIMA | 467 | Mariquita |
| TOLIMA | 468 | Carmen de Apicalá, Melgar |
| TOLIMA | 469 | Natagaima |
| TOLIMA | 470 | Ortega |
| TOLIMA | 471 | Planadas |
| TOLIMA | 472 | Purificación, Suárez |
| TOLIMA | 473 | Rioblanco |
| TOLIMA | 474 | Cajamarca |
| TOLIMA | 475 | Rovira |
| TOLIMA | 476 | Casabianca, Herveo, Falan, Palocabildo |
| TOLIMA | 477 | Murillo, Villahermosa, Santa Isabel |
| TOLIMA | 478 | Ambalema, Armero |
| TOLIMA | 479 | Alvarado, Coello, Piedras, Anzoategui |
| TOLIMA | 480 | Valle de San Juan, San Luis, Saldaña |
| TOLIMA | 481 | Roncesvalles, San Antonio |
| TOLIMA | 482 | Cunday, Villarrica, Icononzo |
| TOLIMA | 483 | Alpujarra, Prado, Dolores |
| VALLE | 484 | Cali |
| VALLE | 485 | Andalucía |
| VALLE | 486 | Ansermanuevo, El Aguila |
| VALLE | 487 | Buenaventura |
| VALLE | 488 | Buga |
| VALLE | 489 | Bugalagrande |
| VALLE | 490 | Caicedonia |
| VALLE | 491 | Candelaria |
| VALLE | 492 | Alcalá, Cartago, Ulloa |
| VALLE | 493 | Dagua, La Cumbre |
| VALLE | 494 | El Cerrito |
| VALLE | 495 | Florida |
| VALLE | 496 | Guacari, Ginebra |
| VALLE | 497 | Jamundí |
| VALLE | 498 | La Unión, La Victoria |
| VALLE | 499 | Palmira |
| VALLE | 500 | Pradera |
| VALLE | 501 | Roldanillo |
| VALLE | 502 | Sevilla |

| Department | ID | Admin Name |
|---|---|---|
| VALLE | 503 | Tuluá |
| VALLE | 504 | Yumbo |
| VALLE | 505 | Zarzal |
| VALLE | 506 | Argelia, Versalles, El Cairo |
| VALLE | 507 | Obando, Toro |
| VALLE | 508 | Bolívar, El Dovio |
| VALLE | 509 | Riofrío, Trujillo |
| VALLE | 510 | San Pedro, Yotoco |
| VALLE | 511 | Darién (Calima), Vijes, Restrepo |
| ARAUCA | 512 | Arauca, Cravo Norte |
| ARAUCA | 513 | Arauquita, Fortul |
| ARAUCA | 514 | Saravena |
| ARAUCA | 515 | Puerto Rondón, Tame |
| CASANARE | 516 | Yopal |
| CASANARE | 517 | Hato Corozal, Paz de Ariporo |
| CASANARE | 518 | La Salina, Pore, Támara, Nunchía, Trinidad, Sácama |
| CASANARE | 519 | Aguazul, Maní, Orocué, Recetor, San Luis de Palenque |
| CASANARE and VICHADA | 520 | Chameza, Monterrey, Sabanalarga, Tauramena, Villanueva, Puerto Carreño, La Primavera, Santa Rita, Santa Rosalia, San José de Ocune, Cumbaribo |
| PUTUMAYO | 521 | Mocoa |
| PUTUMAYO | 522 | Orito |
| PUTUMAYO | 523 | Puerto Asis |
| PUTUMAYO | 524 | Valle del Guamuéz, San Miguel |
| PUTUMAYO | 525 | Colón, Sibundoy, San Francisco, Santiago, Villagarzón, Puerto Caicedo |
| PUTUMAYO | 526 | Villagarzón, Puerto Caicedo |
| PUTUMAYO | 527 | Puerto Guzmán, Puerto Leguízamo |
| SAN ANDRES | 528 | San Andrés, Providencia |
| AMAZONAS | 529 | Leticia, El Encanto, La Chorrera, Puerto Arica, La Pedrera, Mirití-Paraná, Puerto Nariño, Puerto Santander, Tarapacáá |
| GUAINA | 530 | Inírida, Barranco Mina, San Felipe, Puerto Colombia, La Guadalupe, Cacahual, Pana Pana, Morichal |
| GUAVIARE | 531 | San José del Guaviare |
| VAUPES | 532 | Mitú, Caruru, Pacoa, Taraira, Papunahua, Yavaraté |
| GUAVIARE and VAUPES | 533 | Calamar, El Retorno, Miraflores |
