# Supplementary material for: Modeling human migration across spatial scales in Colombia
Source: PLoS One. 2020 May 7;15(5):e0232702. doi: 10.1371/journal.pone.0232702 (PMC7205305; doi:10.1371/journal.pone.0232702)
Supplement: S3 Table — (PDF) [file pone.0232702.s006.pdf]

S3 Table. Coefficients of the best model under the broad-scale modeling approach

Covariate		Median	95% CI*	R-hat
**Distance between origin and destination**	$DIST_{IJ}$	-2.628	[-2.655, -2.598]	1.000
**Population of origin**	POP_I	-0.147	[-0.165, -0.129]	1.000
**Population of destination**	POP_J	0.373	[0.353, 0.392]	1.000
**Contiguity of origin and destination**	$CONT_{IJ}$	0.703	[0.689, 0.717]	1.000
**Urban proportion of origin**	$URBANPROP_I$	0.517	[0.5, 0.535]	1.000
**Urban proportion of destination**	$URBANPROP_J$	0.608	[0.592, 0.625]	1.000
**Percentile of the origin population**	$PERC_I$	-1.016	[-1.042, -0.988]	1.000
**Percentile of the destination population**	$PERC_J$	0.718	[0.693, 0.745]	1.000
**Population of destination <10th percentile**	$TINY_J$	1.127	[1.06, 1.198]	1.000
**Average per-capita gross cell product of destination**	$GECON_J$	0.265	[0.246, 0.283]	1.000
**Intercept**		-7.938	[-7.954, -7.922]	1.000

* Credible Intervals (CI) obtained from the 2.5% and 97.5% quantiles of each parameter's distribution.
