# Supplementary material for: Modeling human migration across spatial scales in Colombia
Source: PLoS One. 2020 May 7;15(5):e0232702. doi: 10.1371/journal.pone.0232702 (PMC7205305; doi:10.1371/journal.pone.0232702)
Supplement: S4 Table — (PDF) [file pone.0232702.s007.pdf]

S4 Table. Coefficients of the best model under the intermediate-scale modeling approach

Covariate		Median	95% CI*	R-hat
**Distance between origin and destination**	$DIST_{IJ}$	-3.18	[-3.197, -3.16]	1.000
**Population of origin**	POP_I	-0.092	[-0.095, -0.089]	1.000
**Population of destination**	POP_J	0.305	[0.301, 0.309]	1.000
**Contiguity of origin and destination**	$CONT_{IJ}$	1.449	[1.438, 1.461]	1.000
**Urban proportion of origin**	$URBANPROP_I$	0.67	[0.664, 0.677]	1.000
**Urban proportion of destination**	$URBANPROP_J$	-0.513	[-0.522, -0.504]	1.000
**Percentile of the destination population**	$PERC_J$	1.048	[1.035, 1.061]	1.000
**Population of origin > 90th percentile**	$MAJCEN_I$	-0.421	[-0.432, -0.411]	1.000
**Population of destination > 90th percentile**	$MAJCEN_J$	0.765	[0.752, 0.776]	1.000
**Intercept**		-9.84	[-9.851, -9.829]	1.000

* Credible Intervals (CI) obtained from the 2.5% and 97.5% quantiles of each parameter's distribution.
